# A global genotyping survey of *Strongyloides stercoralis* and *Strongyloides fuelleborni* using deep amplicon sequencing

**DOI:** 10.1371/journal.pntd.0007609

**Published:** 2019-09-16

**Authors:** Joel L. N. Barratt, Meredith Lane, Emir Talundzic, Travis Richins, Gemma Robertson, Fabio Formenti, Bobbi Pritt, Guilherme Verocai, Joelma Nascimento de Souza, Neci Mato Soares, Rebecca Traub, Dora Buonfrate, Richard S. Bradbury

**Affiliations:** 1 Division of Parasitic Diseases and Malaria, Parasitic Diseases Branch, Centers for Disease Control and Prevention, Atlanta, Georgia, United States of America; 2 Oak Ridge Associated Universities, Oak Ridge, Tennessee, United States of America; 3 Synergy America Inc., Atlanta, Georgia, United States of America; 4 Franklin College of Arts and Sciences, University of Georgia, Athens, Georgia, United States of America; 5 Rollins School of Public Health, Emory University, Atlanta, Georgia, United States of America; 6 Forensic and Scientific Services, Health Support Queensland, Brisbane, Queensland, Australia; 7 Department of Infectious–Tropical Diseases and Microbiology, IRCCS Sacro Cuore Don Calabria Hospital, Negrar, Verona, Italy; 8 Mayo Clinic, Rochester, Minnesota, United States of America; 9 College of Veterinary Medicine and Biomedical Sciences, Texas A&M University, Texas, United States of America; 10 Faculdade de Farmacia, Universidade Federal da Bahia, Bahia, Brazil; 11 Faculty of Veterinary Science, University of Melbourne, Victoria, Australia; Consejo Nacional de Investigaciones Cientificas y Tecnicas, Fundación Mundo Sano, ARGENTINA

## Abstract

Strongyloidiasis is a neglected tropical disease caused by the human infective nematodes *Strongyloides stercoralis*, *Strongyloides fuelleborni fuelleborni* and *Strongyloides fuelleborni kellyi*. Previous large-scale studies exploring the genetic diversity of this important genus have focused on Southeast Asia, with a small number of isolates from the USA, Switzerland, Australia and several African countries having been genotyped. Consequently, little is known about the global distribution of geographic sub-variants of these nematodes and the genetic diversity that exists within the genus *Strongyloides* generally. We extracted DNA from human, dog and primate feces containing *Strongyloides*, collected from several countries representing all inhabited continents. Using a genotyping assay adapted for deep amplicon sequencing on the Illumina MiSeq platform, we sequenced the hyper-variable I and hyper-variable IV regions of the *Strongyloides* 18S rRNA gene and a fragment of the mitochondrial cytochrome c oxidase subunit 1 (*cox1*) gene from these specimens. We report several novel findings including unique *S*. *stercoralis* and *S*. *fuelleborni* genotypes, and the first identifications of a previously unknown *S*. *fuelleborni* infecting humans within Australia. We expand on an existing *Strongyloides* genotyping scheme to accommodate *S*. *fuelleborni* and these novel genotypes. In doing so, we compare our data to all 18S and *cox1* sequences of *S*. *fuelleborni* and *S*. *stercoralis* available in GenBank (to our knowledge), that overlap with the sequences generated using our approach. As this analysis represents more than 1,000 sequences collected from diverse hosts and locations, representing all inhabited continents, it allows a truly global understanding of the population genetic structure of the *Strongyloides* species infecting humans, non-human primates, and domestic dogs.

## Author summary

Strongyloidiasis is a neglected tropical disease caused by the human infective worms (nematodes) *Strongyloides stercoralis*, *Strongyloides fuelleborni fuelleborni* and *Strongyloides fuelleborni kellyi*. Little is known about the genetic diversity of these nematodes and the possibility of geographically isolated genetic types is a particularly interesting research question, given the unique life cycle of these worms which includes both sexual and asexual stages. We extracted DNA from human, dog and primate feces containing *Strongyloides*, collected from several countries representing all inhabited continents. Using next-generation sequencing, we analyzed three *Strongyloides* DNA fragments from these specimens; two fragments of the 18S gene and one from the cytochrome c oxidase subunit 1 (*cox1*) gene. Using this approach, we discovered some unique *S*. *stercoralis* and *S*. *fuelleborni* genotypes, and identified a previously unknown *S*. *fuelleborni* infecting humans within Australia. We compared our data to all 18S and *cox1* sequences of *S*. *fuelleborni* and *S*. *stercoralis* available in public databases and identified several patterns relating to the global distribution of certain genotypes. This knowledge could allow us to infer the origin of human *Strongyloides* infections in the future, and assess the role certain animals (non-human primates and dogs) might play in the transmission of *Strongyloides* to humans.

## Introduction

The genus *Strongyloides* (nematoda: Rhabditida) contains at least fifty different species of parasitic nematodes [1], often showing remarkable host specificity [2]. Humans are most commonly infected with *Strongyloides stercoralis*, which is a soil-transmitted helminth also infecting dogs and non-human primates [1]. Strongyloidiasis is estimated to affect at least 370 million people in over 70 countries [3], mostly within tropical and sub-tropical regions [1]. Under suitable conditions, transmission may also occur in temperate regions [4–6]. Human infection with *Strongyloides fuelleborni fuelleborni* which also infects non-human primates, has also been reported from Southeast Asia [7] and some African countries [8–11]. Another sub-species, *S*. *fuelleborni kellyi* at this time is only known to parasitize humans and is thought to occur exclusively in Papua New Guinea [9, 12, 13]. The majority of these infections are clinically innocuous, even in heavy infections [12]. However, in a small number of infants infected with *S*. *f*. *kellyi*, a syndrome consistent with protein-losing enteropathy occurs. This condition, referred to as “swollen belly” syndrome, has a high fatality rate [12].

Based on examination of a fragment of the 18S (syn. SSU) rRNA gene of each subspecies of *S*. *fuelleborni*, Dorris et al. [14] suggested that *S*. *f*. *kellyi* should be transferred to the species name *Strongyloides kellyi*. Later, Hasegawa et al. [15] examined the 18S rRNA gene of multiple species of *Strongyloides*, including *S*. *stercoralis* (five from dogs, two from humans and one from a chimpanzee) and *S*. *f*. *fuelleborni* (six of non-human primate origin, and one from a human in Africa). This study also introduced a genotyping scheme based on nucleotide variants at the hyper-variable regions (HVRs) of the 18S rRNA gene, describing variations in HVRs I-IV. The same group later described HVR-IV and cytochrome c oxidase subunit 1 (*cox1*) haplotypes of multiple *Strongyloides* spp., including *S*. *f*. *fuelleborni* and *S*. *stercoralis*, from humans and non-human primates, predominantly from Japan and Africa [16]. This work was later expanded upon by several other investigators who continued to utilize *cox1* in conjunction with the 18S HVR-I and 18S HVR-IV loci as the preferred targets for genotyping *S*. *stercoralis* [17–22].

These previous efforts to genotype *S*. *stercoralis* and *S*. *fuelleborni* involved the time consuming, costly, and difficult process of culture and DNA extraction of multiple individual larvae from each host to be tested [15, 17–20, 22]. This approach was required because in mixed genotype infections, chain-termination (Sanger) sequencing might only detect the most prevalent genotype, or generate mixed sequence chromatograms due to the presence of multiple genotypes, resulting in dual peaks that can be difficult to interpret. Furthermore, in cases where indels of differing lengths occur in the same amplicon, the chromatograms may be uninterpretable. Earlier studies have demonstrated that a single host may be infected with multiple *Strongyloides* spp. genotypes [17, 20]. We considered that next-generation sequencing could be used to address some of these challenges, allowing investigators to genotype infections directly from DNA extracted from stool.

In this paper, we describe a novel next-generation sequencing-based method for genotyping nematodes of the Strongyloididae family that employs three PCR assays targeting the informative 18S HVR-I, 18S HVR-IV and *cox1* loci. We use this assay to test the hypothesis that additional, previously undetected genotypes of *Strongyloides* spp. are present globally, and provide some preliminary information on the geographic diversity of known and novel genotypes. To assist in clarity and standardization of *Strongyloides* spp. genotype nomenclature into the future, we also expand an existing *Strongyloides* spp. genotyping scheme to accommodate these novel types.

## Methods

### Fecal sample collection and initial detection of *Strongyloides* species

Human, domestic dog and non-human primate fecal samples found positive for *Strongyloides* species and preserved in ethanol, or fecal DNA extracts from frozen stool were collected from various global locations. All human and dog samples were anonymized post-diagnostic specimens, with the exception of *S*. *stercoralis* strain PV001 strain, which is maintained at the University of Pennsylvania and was kindly provided courtesy of Dr. Thomas J. Nolan, University of Pennsylvania. Human samples were collected directly into specimen containers by patients for diagnostic purposes, domestic dog samples were obtained from the ground after defecation. The Gambian baboon sample was from feces collected from the ground in the vicinity of the infected host. Multiple methods were used to confirm *Strongyloides* infection: real-time PCR [23], Koga agar plate culture [24], direct microscopy and formalin-ethyl acetate microscopy. Samples were transported to the Centers for Disease Control and Prevention for analysis either at room temperature (ethanol preserved feces) or on dry ice (DNA extracts and frozen samples). Ethanol preserved and fresh-frozen samples were extracted upon receipt and all DNA extracts were stored at -20°C until analysis.

### DNA extraction

Genomic DNA was extracted from stool samples collected from Africa, Europe and Asia (excluding Cambodia) as previously described [25]. Briefly, this involved the use of the MagNA Pure LC 2.0 Instrument (Roche Diagnostics), following the DNA I Blood Cells High performance II protocol, utilizing DNA isolation kit I (Roche Diagnostics). The samples from Cambodia and samples from all other regions were centrifuged at 1500 g for one minute, followed by resuspension in sterile saline solution and storage at 4°C overnight to remove excess ethanol and inhibitors. Following this, the samples were washed by centrifugation at 1500 g for one minute and resuspended in sterile saline. This suspension was immediately centrifuged a third time at 1500 g for one minute and the pellet used for isolation of DNA. DNA was extracted using a Qiagen DNeasy Power Soil DNA isolation kit (Qiagen, Germantown, MD) following the manufactures instructions, but with only a sixty second bead beating stage. Extracted DNA was stored at -20°C.

### Assay design and optimization

The nuclear *18S rRNA* (HVR-I and HVR-IV) and mitochondrial *cox1* assays were designed specifically for adaptation to the Illumina sequencing platform, utilizing paired-end reads with a read length of 250 base pairs. To simplify data analysis and increase assay sensitivity, the amplicons were kept relatively short (a maximum of ~450 base pairs). This would facilitate the generation of paired reads that overlap and span the entire length of the amplicon after quality/adapter trimming and merging. For the *cox1* region, priming sites were selected that; (1) resolve the two known *S*. *stercoralis* lineages (groups A and B) previously described into different clusters [19, 20], (2) fulfill the criteria for amplicon length (<450 base pairs), and (3) potentially detect and differentiate strongylid nematodes (i.e. hookworms) as well as various *Strongyloides* species. All primers were designed manually using Geneious Primer design software (version 11); their sequences are shown in Table 1. Each of the three reactions was optimized by testing amplification performance across a range of annealing temperatures, guided by the NEB *T*_*m*_ calculator (https://tmcalculator.neb.com/#!/main), and by testing different reaction volumes and additives (e.g. High GC Enhancer). Following agarose gel electrophoresis, reaction conditions that yielded a clean, bright band reflecting high target DNA yield in the absence of spurious bands, were considered optimal. The optimal conditions for each assay are described below.

**Table 1:** PCR primers and reaction conditions.

Primer	Amplicon Length ^α^	Sequence	Reaction conditions	Sequencing success ^γ^
SSP_COX1_F * SSP_COX1_R *	217 bp	5’-TTTGATCCTAGTTCTGGTGGTAATCC-3’ 5’-GTAGCAGCAGTAAAATAAGCACGAGA-3’	**Step 1:** 98°C for 30 s, **Step 2:** 98°C for 10 s, **Step 3:** 60°C for 10 s, **Step 4:** 72°C for 10 s, **Step 5:** 72°C for 2 min. Repeat steps two to four 45 times.	38/60 (63%)
NEW_HVR_I_F NEW_HVR_I_R	~ 434 bp ^β^	5’-GCTCATTATAACAGCTATAGACTACACGGTA-3’ 5’-CCACAACAATCATTTTATGCACTTGG-3’	**Step 1:** 98°C for 30 s, **Step 2:** 98°C for 10 s, **Step 3:** 60°C for 10 s, **Step 4:** 72°C for 10 s, **Step 5:** 72°C for 2 min. Repeat steps two to four 45 times.	48/60 (80%)
NEW_HVR_IV_F NEW_HVR_IV_R	~ 255 bp ^β^	5’-CGGGCCGGACACTATAAGG-3’ 5’-ATCTCTAAACAGGAACATAATGATCACTAC-3’	**Step 1:** 98°C for 30 s, **Step 2:** 98°C for 10 s, **Step 3:** 63°C for 10 s, **Step 4:** 72°C for 10 s, **Step 5:** 72°C for 2 min. Repeat steps two to four 45 times.	41/60 (68%)

^α^ After removing PCR primer sequences from the amplicon

^β^ Length varies depending on haplotype

^γ^ Based on the number of successfully genotyped specimens at this locus from a total of 60 specimens.

*Broadly specific primers for generation of *Strongyloides* spp. *cox*1 amplicons as well as those of several strongylids.

### PCR amplification

For the *cox1* locus, reactions were performed in a volume of 50 μL using reagents provided with the Q5 High-Fidelity DNA Polymerase (New England Biolabs, Ipswich, MA), including 10 μL NEB 5X Q5 Buffer (New England BioLabs, USA), 10 μL NEB 5X Q5 High GC Enhancer (New England BioLabs, USA), 4 μL NEB Deoxynucleotide Solution Mix (10 mM each nt) (New England BioLabs, USA), 1 μL Q5 High-Fidelity DNA Polymerase (New England BioLabs, USA), 2.5 μL forward primer (SSP_COX1_F), 2.5 μL reverse primer (SSP_COX1_R), 18 μL deionized H_2_O, and 2 μL DNA template. For the HVR-I and HVR-IV regions, PCRs were performed in a 25 μL reaction also using reagents provided with the NEBNext Q5 Hot Start kit, including 12.5 μL of HiFI PCR Mastermix (New England BioLabs, USA), 1.5 μL forward primer (NEW_HVR_I_F or NEW_HVR_IV_F), 1.5 μL reverse primer (NEW_HVR_I_R or NEW_HVR_IV_R), 7.5 μL deionized H_2_O, and 2 μL DNA template. Each PCR run was accompanied by a positive control consisting of genomic DNA from *S*. *stercoralis* PV001 strain, a negative feces DNA extract control and a PCR grade water negative control. The *S*. *stercoralis* PV001 strain positive control further served as an internal control for sequencing analysis of samples (i.e. sequenced multiple times to ensure lack of errors), as did a *Strongyloides ratti* control which served as a control for cross contamination at any step [21]. The reaction was performed on a GeneAmp 9700 thermocycler (Applied Biosystems, Beverly, MA), using the temperature cycling conditions provided in in Table 1.

### Illumina sequencing

PCR products of the correct fragment size on agarose gel electrophoresis were prepared for deep-sequencing. Amplicons were purified and normalized using the SequalPrep Normalization Plate Kit (Thermo Fisher Scientific, Waltham, MA) and DNA library preparation performed using the NEBNext Ultra DNA Library Prep Kit for Illumina (New England BioLabs, Ipswich, MA). Library indices were added using the NEBNext Mupltiplex Oligos for Illumina Index kit (New England BioLabs, Ipswich, MA). Sequencing was performed using the Illumina MiSeq platform with MiSeq reagent Nano Kit v2 (PE250bp) reagent kits (Illumina, San Diego, CA).

### Bioinformatic analysis

Bioinformatic analysis of all sequence data was undertaken using a custom workflow designed in Geneious (Geneious Prime, version 11: www.geneious.com). This workflow performed read quality control, assembly of contigs and 18S HVR-I and HVR-IV haplotype assignment after Jaleta et al. [19], and included the adjustments to that typing scheme that we previously introduced [21]. As the sequence of *cox1* is extremely variable, with hundreds of haplotypes, and because our *cox1* amplicon is substantially shorter than those previously described, we did not assign haplotypes to our *cox1* sequences. This was done to avoid confusion with other studies. Instead, *cox1* sequences were assigned to clusters that were visualized by construction of cluster dendrograms with various clusters assigned to colors. Additionally, each *cox1* sequence can be uniquely identified by the GenBank (GB) accession numbers assigned to them. For generation of cluster dendrograms, a .fasta sequence file containing all *cox1* sequences was exported from Geneious and aligned using the ‘msa’ package in R (https://www.r-project.org/). Using the ‘seqinr’ package ‘dist.alignment’ function, a pairwise identity matrix was constructed, considering gaps in the identity measure. Clustering was then performed using the agglomerative nested clustering approach in the ‘agnes’ R package, using euclidean distances and the average clustering method. From this, the ‘ggtree’ R package was used to generate cluster dendrograms. To aid dendrogram annotation, images of relevant hosts were obtained from PhyloPic (http://phylopic.org) or prepared in house at the Centers for Disease Control and Prevention (CDC).

### Ethical clearance

This activity was approved as research not involving human subjects, by the Office of the Associate Director for Science, Center for Global Health, at CDC. Because the study did not involve direct interaction with animals, Animal Care and Use committee approval was not required (Protocol number 2017–535).

## Results

### Expansion of the *Strongyloides* spp. typing scheme to accommodate novel *S*. *stercoralis* haplotypes and *S*. *fuelleborni*

**Fig 1:**
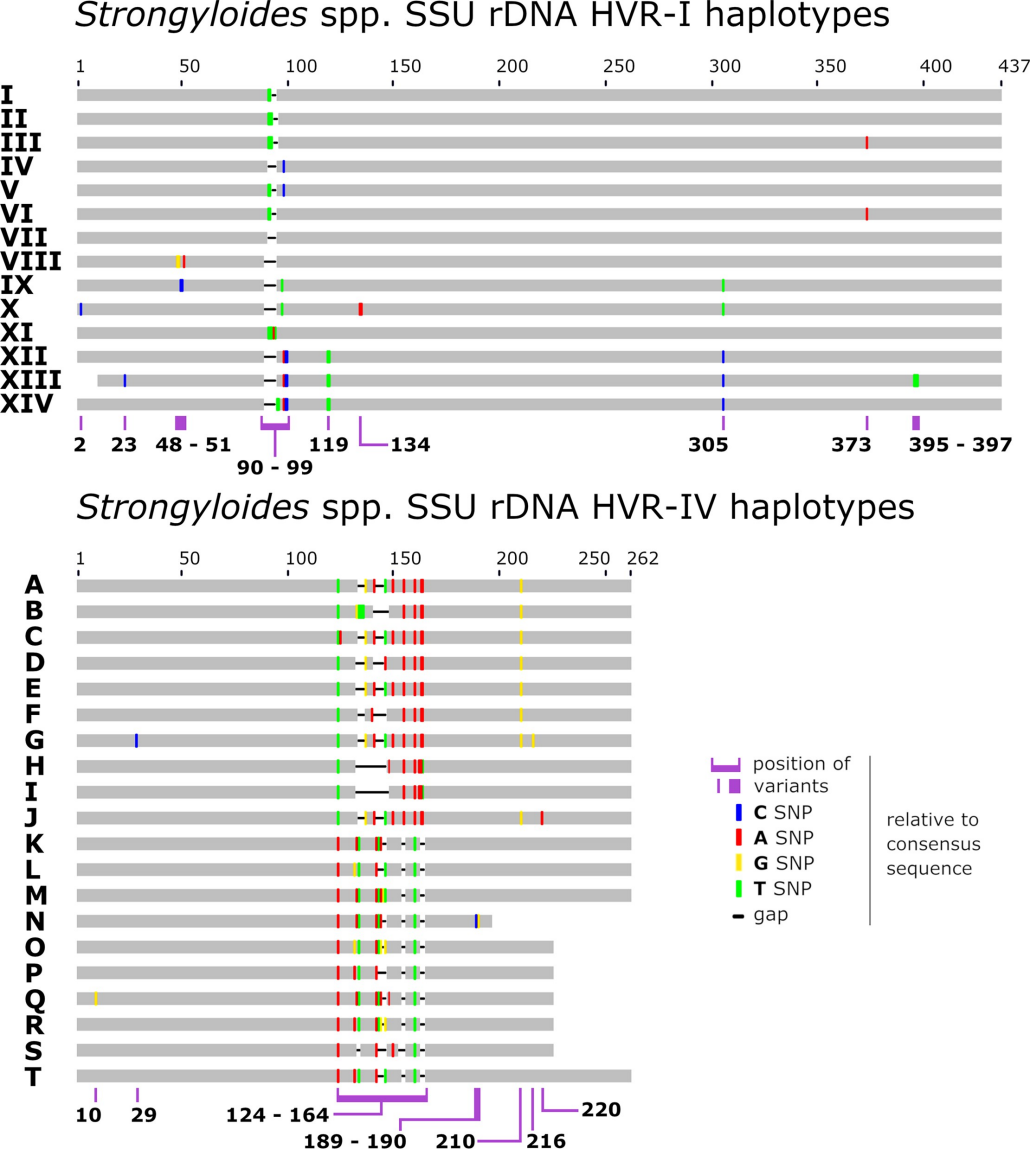
Schematic of the *Strongyloides* spp. genotyping scheme. A graphical representation of the *Strongyloides* spp. genotyping scheme described previously [21], expanded to include additional genotypes from *S*. *stercoralis* and *S*. *fuelleborni*. This scheme includes novel sequences identified in this study and 18S sequences available in GenBank (GB), where the appropriate 18S HVR-I and/or HVR-IV regions were captured. Sequences from GB possessing Ns or ambiguous bases were excluded from the scheme. For additional details on the hosts in which these *Strongyloides* spp. haplotypes were detected, refer to Tables 2 and 3.

We employed this assay previously to screen feces from Australian dogs and humans [21] and identified multiple cryptic *Strongyloides* spp. genotypes in some dogs, leading us to propose several adjustments to the *Strongyloides* spp. genotyping scheme established by previous investigators [16, 17, 19]. We justified these adjustments [21] by highlighting two important points: (1) the cryptic *Strongyloides* spp. sequences we described were identified within feces of the same host as previously described *S*. *stercoralis* genotypes; domestic dogs, and (2), the cryptic *Strongyloides* spp. sequences added to the scheme were more similar to each other than to *S*. *ratti* [21]. It was noted that some of these cryptic *Strongyloides* spp. genotypes may have been present in the dogs due to coprophagy, though given that this is speculative these types were added to the scheme nonetheless [21]. This was considered a straightforward solution to the problem of assigning sequences an identity, thereby facilitating ease of comparison to known haplotypes and subsequent discussion. In line with these principles, we adjusted the typing scheme further in this study to include all 18S HVR-I and HVR-IV haplotypes shown in Fig 1, and we list published examples of these sequences (Table 2 and Table 3).

**Table 2:** HVR-I haplotypes assigned to *Strongyloides* spp. as part of this typing scheme.

Haplotypes	GenBank Accession/s	*Strongyloides* sp/spp. assignment	Hosts
I	AB923888.1, AF279916.2, AJ417023.1, KF926658.1, MN076327—MN076334	*Strongyloides stercoralis*	dog and human
II	KF926659.1, MK468654, MK468655, MK468656—MK468658, MN076335—MN076356	*Strongyloides stercoralis*	dog and human
III	AB453315.1, KF926660.1, MN076357—MN076360	*Strongyloides stercoralis*	dog and human
IV	AB272234.1, KU724124.1, MK468659, MK468663	*Strongyloides stercoralis* and *Strongyloides procyonis*	*Strongyloides stercoralis* dog genotype and *S*. *procyonis* genotype found in a badger
V	MN076361	*Strongyloides stercoralis*	dog
VI	AB453316.1, AB453314.1, MH932098.1, MH932099.1, MH932100.1, MK468660, MN076362—MN076374	*Strongyloides stercoralis*	human, dog, and chimpanzee
VII	AB205054.1	*Strongyloides procyonis*	racoons
VIII	MK468661	Cryptic *Strongyloides* sp.	dog
IX	LC038066.1	*Strongyloides* sp. Okayama	Japanese striped snake
X	MK468662	Cryptic *Strongyloides* sp.	dog ^α^
XI *	MN076375, MN076376	*Strongyloides stercoralis*	human
XII	AB453320.1, AB453321.1, AB821045.1, AB821046.1, MN076377—MN076380	*Strongyloides fuelleborni*	gorillas, chimpanzees, humans and baboons
XIII	AB453322.1	*Strongyloides fuelleborni*	gorilla
XIV	AB677955.1, AB272235.1, AB453317.1, AB453318.1, AB453319.1	*Strongyloides fuelleborni*	Japanese macaque (*Macaca fuscata*).

^α^ Suspected to be the result of coprophagy (reptile feces) or ingestion of reptiles as this type is most similar to *Strongyloides* sp. Okayama.

* Novel haplotype detected in this study

**Table 3:** HVR-IV haplotypes assigned to *Strongyloides* spp. as part of this typing scheme.

Haplotypes	GenBank Accession/s	*Strongyloides* sp/spp. assignment	Host
A	AB526827.1, KY081221.1, KU962182.1, KU962181.1, KU962180.1, KU962179.1, KY081223.1, AB526826.1, AB453316.1, AB453315.1, AB453314.1, MK468664—MK468671, MN076381, MN076382, MN076384—MN076394, MN076396—MN076406, MN076409—MN076414, MN076416—MN076423	*Strongyloides stercoralis*	dog, human and chimpanzee
A(i)[A_1454] ^β^	KF926662.1, KU724125.1, KU724128.1, LC085482.1, AF279916.2	*Strongyloides stercoralis*	dog and human
A(ii)[G_1454] ^β^	KF926661.1, LC085483.1, LC085481.1, AB923888.1, MH932095.1, MH932097.1, MH932096.1	*Strongyloides stercoralis*	dog and human
B	KU724129.1, MK468672, MK468673, MN076383	*Strongyloides stercoralis*	dog
C	M84229.1	*Strongyloides stercoralis*	human from SE Asia
D	AB272234.1, AB205054.1	*Strongyloides procyonis*	badger and raccoon
E ^γ^	MK468674	*Strongyloides stercoralis*	dog
F	MK468675	Cryptic *Strongyloides* sp.	dog
G	MK468676	Cryptic *Strongyloides* sp.	dog
H	LC038066.1	*Strongyloides* sp. Okayama	Japanese striped snake
I	MK468677	Cryptic *Strongyloides* sp.	dog ^α^
J	MN076395*	*Strongyloides stercoralis*	human
K	LC085496.1, LC085493.1, LC085492.1, LC085491.1, LC085484.1, LC085494.1, AB526820.1, AB453320.1, AB526823.1, AB526821.1	*Strongyloides fuelleborni*	chimpanzees and human
L	LC085490.1, LC085489.1	*Strongyloides fuelleborni*	gorilla
M	LC085486.1, MN076407	*Strongyloides fuelleborni*	human
N	LC085497.1	*Strongyloides fuelleborni*	chimpanzee
O	AB453322.1	*Strongyloides fuelleborni*	gorilla
P	LC085488.1, AB526824.1, AB526825.1	*Strongyloides fuelleborni*	chimpanzee and gorilla
Q	AB526822.1	*Strongyloides fuelleborni*	baboon
R	AB453321.1	*Strongyloides fuelleborni*	chimpanzee
S	KY081222.1, AB453317.1, AB453318.1, AB453319.1, AB272235.1, MH045486.1, MH045487.1, MH045487.1, MN076415	*Strongyloides fuelleborni*	human and macaque
T	MN076408*	*Strongyloides fuelleborni*	human

^α^ Suspected to result of coprophagy (reptile feces) or ingestion of reptiles as this type is most similar to *Strongyloides* sp. Okayama.

* Novel haplotype identified in this study

^β^ Schär et al. [17] identified a variant of HVR-IV possessing a “G” at position 1454, while some had an “A” at this position, assigning these to haplotypes G and A respectively. Position 1454 is not captured by the assay described here and this is also true for most sequences generated by Jaleta et al. [19]. All amplicons described by Schar et al. [17] belong to haplotype A as it is defined in this study though an effort was made to differentiate between these two types described by Schar et al. [17] in this table, where possible.

^γ^ This haplotype was defined as haplotype E by Beknazarova et al [21]. When the manuscript by Beknazarova et al. [21] was in press, and following editorial acceptance of the present manuscript, we noted that Zhou et al. [39] had independently assigned a sequence containing our haplotype E variable motif [ATTTGTTTATTTTAATAT] to haplotype C. This is not the same sequence as haplotype C as defined by Beknazarova et al [21] and in the present study, which we assigned to a sequence with the GenBank accession number M84229.1.

### Success of *Strongyloides* spp. genotyping by next generation sequencing

Sequence data was generated for 60 specimens in this study; sequencing success rates for each marker provided in Table 1. One third of these specimens (n = 20) had all markers successfully sequenced, 13 had one marker sequenced and 27 had any two markers sequenced (144 sequences generated in total). Typed specimens were predominantly from humans and domestic dogs (*Canis familiaris*), and were obtained from several countries representing all inhabited continents (Table 4). One typed specimen was from a Guinea baboon (*Papio papio*) from The Gambia and contained *S*. *f*. *fuelleborni*. One human specimen from India contained *S*. *f*. *fuelleborni* and three human specimens from Australia also contained a *S*. *fuelleborni*. Whether this Australian sub-species of *S*. *fuelleborni* is novel or is *S*. *f*. *kellyi* could not be determined using the data available. The remaining *Strongyloides* spp. genotypes detected were attributed to *S*. *stercoralis* (Table 4).

**Table 4:** Human, primate and dog *Strongyloides* spp. specimens analyzed in this study and their genotype.

Specimen Name	Location	Host	HVR-I haplotype	HVR-IV haplotype	*Cox*1 Accessions	Helminth/s detected
Baboon 5_N8_Ga	Gambia	*Papio papio*	XII	-	-	*S*. *fuelleborni*
Dog 1101_It	Italy	Dog	VI	A	MN076424	*S*. *stercoralis*
Dog 1141_It	Italy	Dog	VI	-	MN076425	*S*. *stercoralis*
Dog 4_Ca	Cambodia	Dog	-	A	-	*S*. *stercoralis*
Dog 5_US_Cl_OH	Ohio (USA)	Dog	VI	-	-	*S*. *stercoralis*
Dog 7_Ca	Cambodia	Dog	V	B	-	*S*. *stercoralis*
Dog A4_US_GA	Georgia (USA)	Dog	VI	A	MN076426	*S*. *stercoralis*
Dog A7_US_GA	Georgia (USA)	Dog	VI	A	MN076427	*S*. *stercoralis*
Dog Bu_US_Cl_OH	Cleveland (USA)	Dog	VI	A	MN076428	*S*. *stercoralis*
Dog US_ATL_GA	Georgia (USA)	Dog	VI	-	MN076429	*S*. *stercoralis*
Dog US_GA1	Georgia (USA)	Dog	-	-	MN076430	*S*. *stercoralis*
Dog US_PA	Pennsylvania (USA)	Dog	I and VI	-	MN076431	*S*. *stercoralis*
Dog W2_US_GA	Georgia (USA)	Dog	VI	-	MN076432	*S*. *stercoralis*
Human 1_Ca	Cambodia	Human	-	A	MN076433	*S*. *stercoralis*
Human 1_La	Laos	Human	II	A	MN076459	*S*. *stercoralis*, *Necator americanus*
Human 10_An	Angola	Human	-	A	-	*S*. *stercoralis*
Human 100_Et	Ethiopia	Human	-	A	-	*S*. *stercoralis*
Human 1070_Se	Senegal	Human	-	A	-	*S*. *stercoralis*
Human 10VS_US_KY	Kentucky (USA)	Human	VI	A	-	*S*. *stercoralis*
Human 1207_Br	Bahia (Brazil)	Human	-	A	-	*S*. *stercoralis*
Human 14WC_US_LA	Louisiana (USA)	Human	II and VI	A and J	MN076434	*S*. *stercoralis*
Human 1540_Ni	Nigeria	Human	II	A	MN076451	*S*. *stercoralis*
Human 1598_Gu_Co	Guinea (Conakry)	Human	II	A	MN076452	*S*. *stercoralis*
Human 169_Et	Ethiopia	Human	I and II	A	MN076438	*S*. *stercoralis*
Human 2_Ca	Cambodia	Human	II	NA	MN076460	*S*. *stercoralis*, *N*. *americanus*
Human 21_17_La	Laos	Human	VI	-	MN076435	*S*. *stercoralis*
Human 229_Au	Queensland (Australia)	Human	I	-	MN076439	*S*. *stercoralis*
Human 238_Au	Queensland (Australia)	Human	II	A	MN076440	*S*. *stercoralis*
Human 25_17_La	Laos	Human	VI	A	-	*S*. *stercoralis*
Human 3_Ca	Cambodia	Human	II	A	-	*S*. *stercoralis*
Human 308_Au	Queensland (Australia)	Human	II	A	MN076441	*S*. *stercoralis*
Human 333_Au	Queensland (Australia)	Human	XII	-	MN076442, MN076461	*S*. *fuelleborni*, *N*. *americanus*
Human 349_7_Au	Queensland (Australia)	Human	II	A	MN076443	*S*. *stercoralis*
Human 352_Au	Queensland (Australia)	Human	-	A	-	*S*. *stercoralis*
Human 358_Au	Queensland (Australia)	Human	-	A	-	*S*. *stercoralis*
Human 360_Au	Queensland (Australia)	Human	II	-	-	*S*. *stercoralis*
Human 367_Au	Queensland (Australia)	Human	II	A	MN076444	*S*. *stercoralis*
Human 368_16_Au	Queensland (Australia)	Human	XII	M and T	-	*S*. *fuelleborni*
Human 378_Au	Queensland (Australia)	Human	III and XI	A	MN076445, MN076462	*S*. *stercoralis*, *N*. *americanus*
Human 395_Au	Queensland (Australia)	Human	II	A	-	*S*. *stercoralis*
Human 428_Au	Queensland (Australia)	Human	II	A	-	*S*. *stercoralis*
Human 434_Au	Victoria (Australia)	Human	II	-	MN076463, MN076464	*S*. *stercoralis*, *Oesophagostomum* sp.
Human 441_Au	Queensland (Australia)	Human	II	-	MN076446	*S*. *stercoralis*
Human 507_Au	Queensland (Australia)	Human	I	-	MN076447	*S*. *stercoralis*
Human 519_Au	Queensland (Australia)	Human	II	A	-	*S*. *stercoralis*
Human 524_Au	Queensland (Australia)	Human	I	A	MN076448	*S*. *stercoralis*
Human 528_Au	Queensland (Australia)	Human	II	A	-	*S*. *stercoralis*
Human 5325_In	India	Human	-	S	MN076453	*S*. *fuelleborni*
Human 5333_It	Italy	Human	I and III	A	MN076454	*S*. *stercoralis*
Human 5344_Br	Bahia (Brazil)	Human	I	A	MN076455	*S*. *stercoralis*
Human 563_Au	Queensland (Australia)	Human	XII	-	-	*S*. *fuelleborni*
Human 58_It	Italy	Human	III	A	MN076436	*S*. *stercoralis*
Human 588_Au	Queensland (Australia)	Human	II	-	MN076449	*S*. *stercoralis*
Human 877_IvCo	Ivory Coast	Human	XI	A	MN076450	*S*. *stercoralis*
Human 88_GuBi	Guinea-Bissau	Human	-	-	MN076437	*S*. *stercoralis*
Human 930_IvCo	Ivory Coast	Human	II	A	-	*S*. *stercoralis*
Human A3_Br	Bahia (Brazil)	Human	-	A	MN076456	*S*. *stercoralis*
Human A4_Br	Bahia (Brazil)	Human	I and III	A	-	*S*. *stercoralis*
Human A9_Br3	Bahia (Brazil)	Human	II	A	MN076457	*S*. *stercoralis*
Human Et_Au	Western Australia	Human	II	-	MN076458	*S*. *stercoralis*

NA: Specimen excluded due to contamination with our *Strongyloides ratti* control DNA

-: Sequence not obtained due to amplification and/or sequencing failure

Note: GenBank Accession numbers for the HVR-I and HVR-IV sequences and BioSample numbers (for linking each specimen to its associated raw Illumina data) are provided in S1 File.

### Novel *Strongyloides* spp. 18S HVR-I and 18S HVR-IV haplotypes

**Fig 2:**
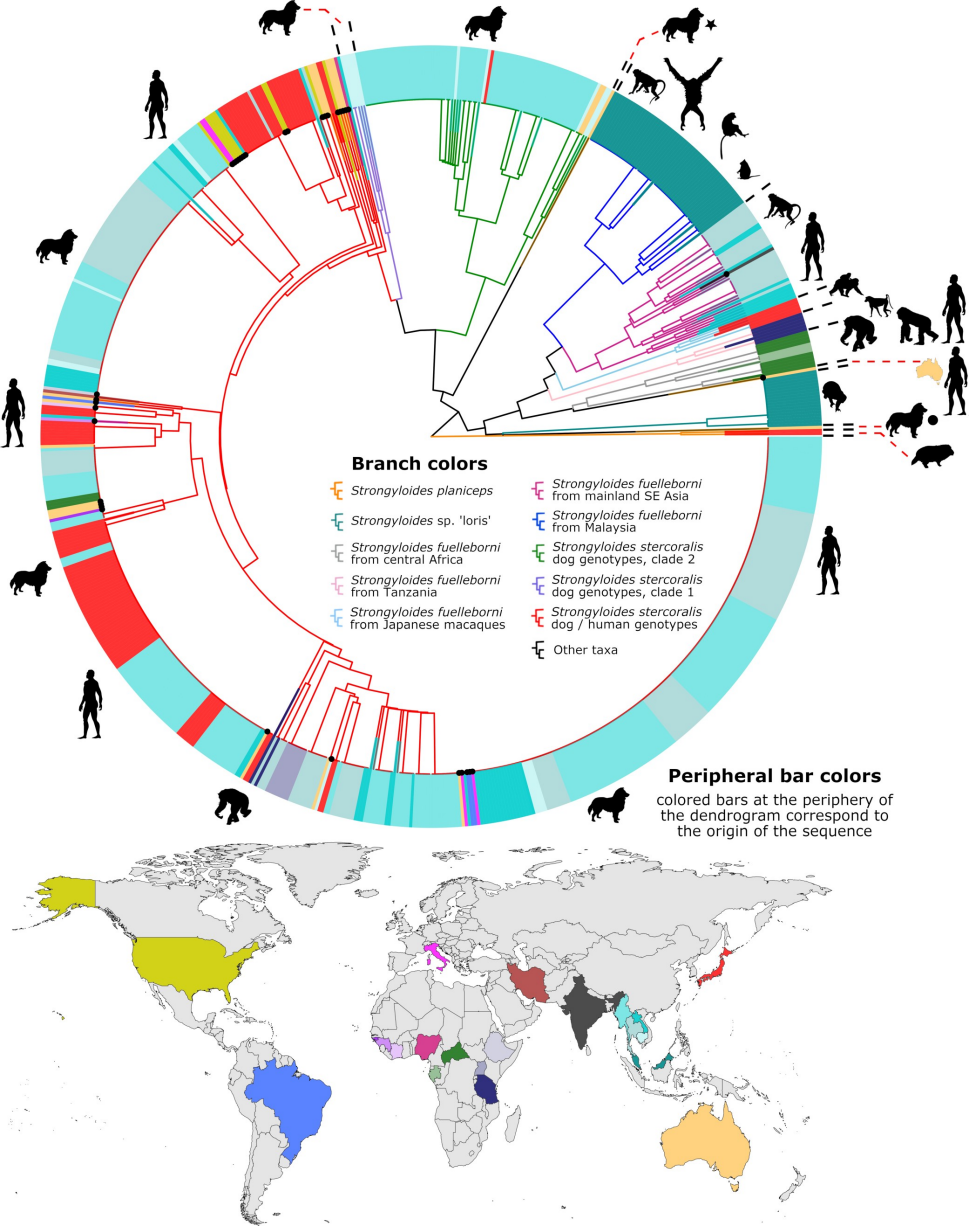
Dendrogram of clustered *cox1* sequences. This dendrogram represents 787 *cox1* sequences, including those generated in this study (branches tipped in a black dot) and all published *cox1* sequences from GB that overlap completely with our 217 base *cox1* amplicon (to our knowledge). Peripheral bars are colored according to their site of origin, which corresponds to the colored countries on the map. Branches are color coded separately, according to their identity; either a species assignment, a genus, or their *S*. *stercoralis* genotype. The dog image with a black star indicates a sequence from an Australian dog generated by us previously [21], that is distinct from other *Strongyloides* spp. and clusters between the *S*. *stercoralis* and *S*. *fuelleborni* groups. The dog image with a black circle highlights a published sequence [21] that clusters close to, yet is distinct from *Strongyloides* spp. detected previously in lorises [27]. Animal images reflect the mammalian hosts that the sequences were associated with. The miniaturized image of Australia next to a human silhouette shows the location of a unique *S*. *fuelleborni cox1* sequence. Two sequences of *Strongyloides planiceps* (orange branches) from Japanese raccoon dogs serve as an outgroup. The identity of each sequence is provided in S1 Fig, which is a searchable PDF of the same dendrogram with all GB accession numbers, the countries of origin, and host species provided. The GB accession numbers for sequences in this dendrogram that were generated as part of this study (branches tipped in a black dot) are provided in S1 File. The sequences used to construct this dendrogram are provided in S2 File.

**Fig 3:**
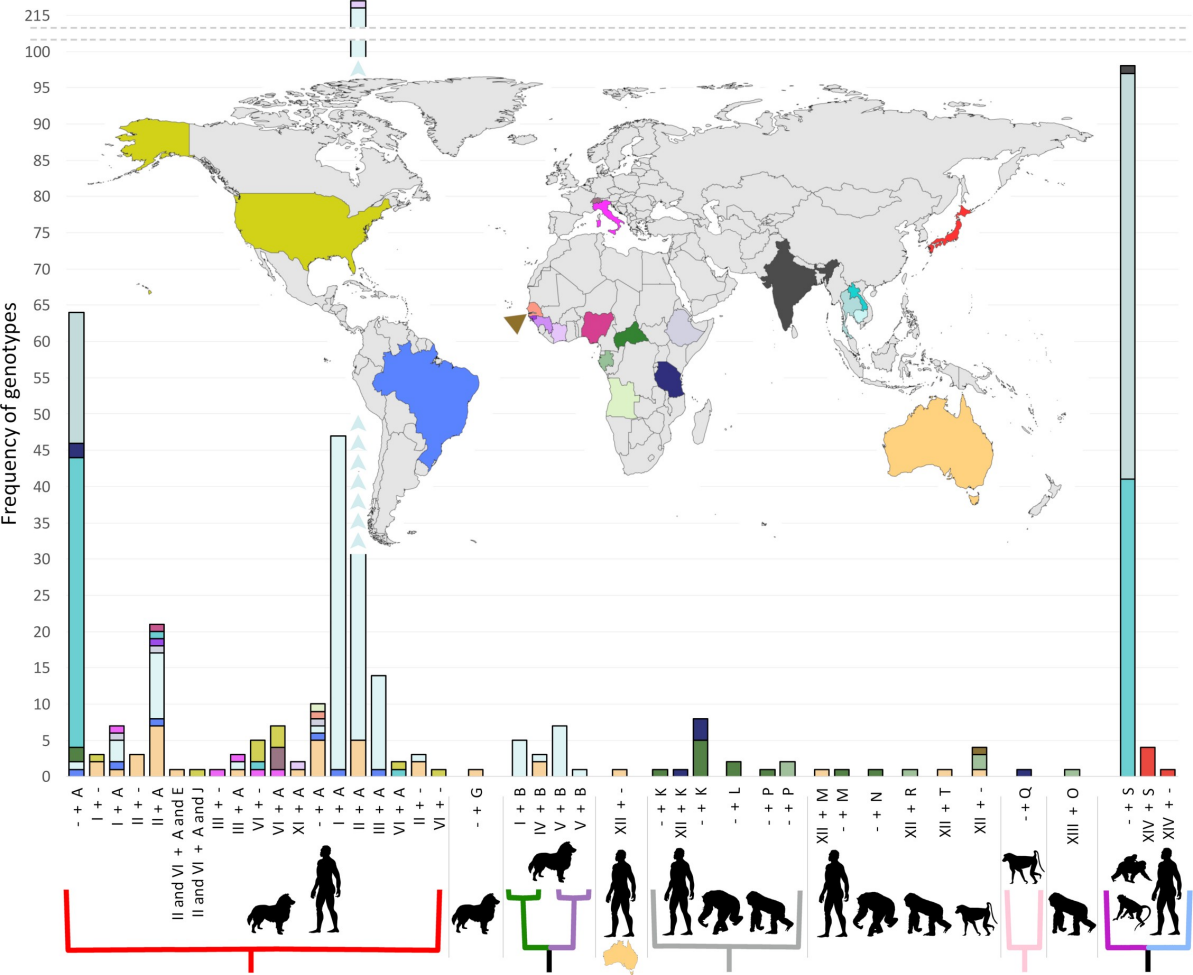
Global frequency of *Strongyloides* spp. 18S genotypes. Histogram bars are colored according to their origin, corresponding to the colors on the map. A dash (-) in the horizontal axis labels indicates a missing 18S haplotype (either HVR-I or HVR-IV). The colored branches below the horizontal axis correspond to the colored branches in Fig 2, where red represents *S*. *stercoralis* types infecting both dogs and humans (lineage A), the green/purple clade represents *S*. *stercoralis* types infecting only dogs (lineage B), the gray clade represents *S*. *fuelleborni* types from African great apes and humans, the light pink clade represents *S*. *fuelleborni* from a baboon and the magenta/light blue clade represents types found in humans and macaques. The absence of a colored branch under a given group indicates that an associated *cox1* sequence is presently unavailable for these 18S haplotypes. The miniaturized map of Australia under the horizontal axis represents the type associated with a *cox1* sequence that clustered alongside *S*. *fuelleborni* from central Africa, yet is sufficiently distinct to be considered unique.

We describe 18S HVR-IV haplotype J and assign it to *S*. *stercoralis* (Fig 1). This haplotype was found in a human specimen from the USA in association with HVR-I haplotypes II and VI, HVR-IV haplotype A, and a *cox1* sequence belonging to the dog/human infecting lineage (lineage A) of *S*. *stercoralis* (Table 2, Figs 2 and 3, red cluster). We also describe HVR-IV haplotype T, detected in a human specimen from Australia and attributed to an undetermined sub-species of *S*. *fuelleborni*. This sequence was found in conjunction with HVR-I haplotype XII and HVR-IV haplotype M, though sequencing of *cox1* from this specimen was not successful (Table 4). The novel 18S HVR-I haplotype XI is described here for the first time, and is assigned to *S*. *stercoralis* on the basis that its sequence was found twice in association with *cox1* sequences belonging to *S*. *stercoralis* from the dog/human infecting lineage (Fig 2, red cluster) and both times in association with 18S HVR-IV haplotype A (Table 4, Human 378_Au and Human 877_IvCo).

### Clustering of *Strongyloides* spp. *cox1* sequences

Thirty-five *cox1* sequences belonging to *Strongyloides* spp. were generated (plus four from *Necator americanus* and two from *Oesophagostomum* sp.). Clustering of *Strongyloides* spp. *cox1* sequences revealed several trends relating to geography, host preference and associations between certain *cox1* clusters and specific 18S genotypes (Fig 2 and Fig 3). No *cox1* sequences were obtained from dogs possessing HVR-I haplotypes IV and/or V, or HVR-IV haplotype B, which represent exclusively dog-infecting types that correspond to *S*. *stercoralis* lineage/type B as per Nagayasu et al. (2017) [20, 21]. Of the 35 *Strongyloides* spp. *cox1* sequences generated, 33 were obtained from specimens collected from humans and dogs possessing HVR-IV haplotype A. Each of these 33 *cox1* sequences were assigned to the cluster highlighted in red (Figs 2 and 3) containing *S*. *stercoralis* that infect both dogs and humans. This cluster corresponds to *S*. *stercoralis* lineage/type A described by Nagayasu et al. [20]. The remaining 2 *Strongyloides* spp. sequences of these 35 were attributed to *S*. *fuelleborni*. When all published *S*. *fuelleborni* and *S*. *stercoralis cox1* sequences were analyzed alongside our data, several geographic trends emerged relating to the distribution of various *S*. *fuelleborni* types. We note that *S*. *fuelleborni cox1* sequences from Malaysia, mainland SE Asia, Japan, East Africa and Central Africa each form their own distinct clusters (Fig 2; dark blue, magenta, light blue, light pink and gray branch colors, respectively), suggesting that each is a potential geographic and/or host-adapted sub-variant of *S*. *f*. *fuelleborni* (Figs 2 and 3).

### Detection of mixed *Strongyloides* spp. genotypes

We identified seven fecal specimens possessing mixed *Strongyloides* spp. genotypes. One specimen (Human 14WC_US_LA) possessed the genotype II and VI + A and J, attributed to *S*. *stercoralis*. Another from Australia (Human 368_16_Au) possessed the genotype XII + T and M, attributed to *S*. *fuelleborni*. A specimen from the USA (Dog US_PA) contained two haplotypes of *S*. *stercoralis* HVR-I (I and VI) though a HVR-IV genotype was not successfully ascertained. A human specimen from Ethiopia possessed the genotype I and II + A (Human 169_Et), while another from Australia (Human 378_Au) possessed the genotype III and XI + A. A human specimen from Italy (Human 5333_It) and Brazil (Human A4_Br) each possessed the genotype I and III + A (Table 4).

### Identifying mixed infections caused by multiple helminth genera

**Fig 4:**
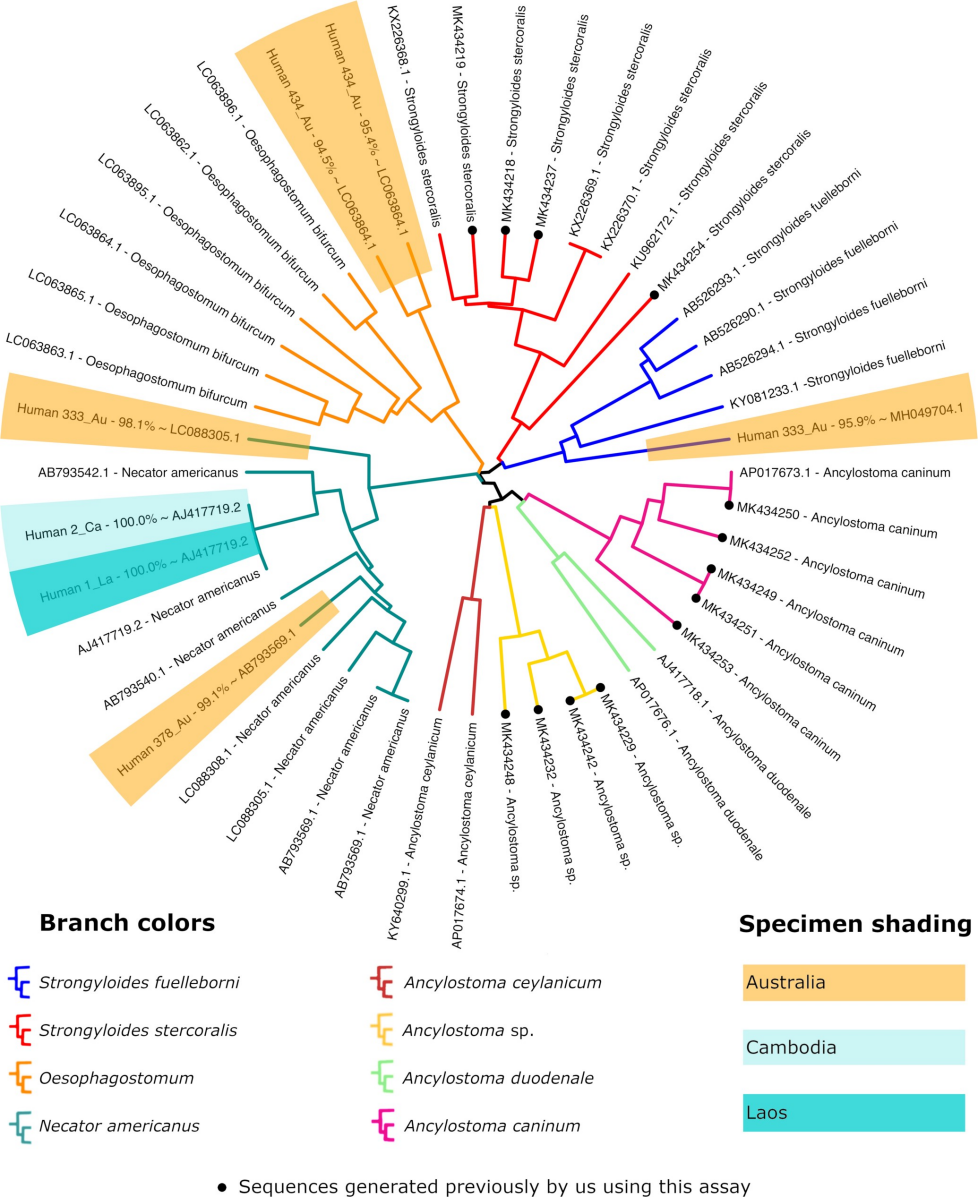
Dendrogram of *cox1* sequences from *Strongyloides* spp. and hookworms. This figure demonstrates that the *cox*1 assay described here is broadly specific for *Strongyloides* spp. and some strongylids. Fragments of *cox1* from *Ancylostoma* spp., *Oesophogostomum* and *Necator americanus* have been amplified and sequenced using this assay. Additionally, we previously detected *cox1* sequences from *Metastrongylus* sp., a rotifer, and some unknown nematodes using this approach [21]. The sequences generated in this study are shaded in colors according to their country of origin and those generated by us in a previous study are marked with a black dot on the associated branch tip. The sequences used to construct this dendrogram are included in S3 File.

The *cox1* assay identified infections caused by multiple helminth species, including members of the genus *Strongyloides* and various strongylids (Fig 4). Also note that we previously reported amplification of *cox1* DNA from *Ancylostoma* sp., as well as *Metastrongylus* sp., possibly some free-living nematodes, and a rotifer (Fig 4) [21], using this assay. A *cox1* amplicon from an Australian specimen (Human 333_Au) contained reads from *S*. *fuelleborni* and *Necator americanus* while another specimen (Human 378_Au) contained DNA from *S*. *stercoralis* and *N*. *americanus*. For other specimens, 18S data were generated for *Strongyloides* spp., though a *cox1* sequence was generated for a strongylid only. In one of these instances where *Strongyloides* 18S data was generated, a *cox1* sequence was detected for *N*. *americanus* only (Human 1_La). Similarly, for another specimen with *Strongyloides* 18S data (Human 434_Au), two *cox1* sequences putatively belonging to an *Oesophogostomum* sp. were obtained (Fig 4). For a third specimen (Human 2_Ca), a sequence was generated for *S*. *stercoralis* 18S HVR-I and while a *cox1* amplicon was generated containing DNA from *N*. *americanus* and *S*. *stercoralis*, the *S*. *stercoralis* reads were too few to generate a contig of sufficient quality.

## Discussion

We used data generated in this study in concert with data published by previous investigators to provide a global understanding of the *Strongyloides* spp. genotypes infecting humans, non-human primates and domestic dogs. An exhaustive search was undertaken to capture all *S*. *stercoralis* and *S*. *fuelleborni* sequences available in GenBank (to our knowledge) for inclusion in this analysis, which resulted in examination of more than 1,000 sequences. This approach provided novel insights on the geographical distribution patterns of *Strongyloides* spp. genotypes, and possible associations between certain genotypes and specific hosts. These observations provide leads for future investigation.

Hasegawa et al. [15] introduced a genotyping scheme including HVRs I-IV of the 18S rRNA gene to study *S*. *stercoralis* from humans and dogs. In 2010, this group introduced *cox1* to aid investigations of genetic diversity within the *Strongyloides* genus [16]. Following this, other investigators continued to sequence various combinations of 18S HVR-I, 18S HVR-IV and *cox1* to characterize *Strongyloides* spp. infecting domestic canines, humans and non-human primates. Studies that represent major contributions to this effort are summarized in Table 5, Fig 2 and Fig 3.

**Table 5:** Human, primate and dog *Strongyloides* spp. specimens analyzed in previous studies and their genotype.

Strain/Isolate name	Country	Host	HVR-I genotype	HVR-IV genotype	*Cox*1 Accession	Reference	Comments
Dog 13	Australia	Dog	-	A and G	-	Beknazarova et al. [21]	NC
Dog 18	Australia	Dog	II and VI	A and E	MK434237		
Dog 22	Australia	Dog	VIII	F	MK434226^α^, MK434227 ^α^		
Dog 32	Australia	Dog	-	A	-		
Dog 45	Australia	Dog	X	I	-		
Dog 6	Australia	Dog	II	A	MK434256		
Dog 6	Australia	Dog	IV	B	MK434255		
Dog 7	Australia	Dog	IV	B	MK434254		
Human 1	Australia	Human	II	A	MK434219		
Human 2	Australia	Human	-	A	-		
Human 3	Australia	Human	II	A	MK434218		
Sputum (Human 4)	Australia	Human	II	A	-		
17D5122	Switzerland	Dog	VI	A(ii)[G_1454]	MH932101.1 ^β^	Basso et al. [30]	NA
18D157	Switzerland	Dog	VI	A(ii)[G_1454]	MH932102.1 ^β^		
18D1644	Switzerland	Dog	VI	A(ii)[G_1454]	MH932103.1 ^β^		
MH045488.1	Laos	Long-tailed macaques	-	S	MH049697.1 to MH049729.1	Thanchomnang et al. [22]	These sequences represent 96 male worms with the “S” genotype– 55 from Thailand and 41 from Laos.
MH045487.1	Thailand	Long-tailed macaques	-	S			
MH045486.1	Thailand	Long-tailed macaques	-	S			
DogKHRovieng-1	Cambodia	Dog	I and II	A	KX226374.1	Jaleta et al. [19]	NC
DogKHRovieng-10	Cambodia	Dog	V	B	KX226383.1		
DogKHRovieng-11	Cambodia	Dog	V	B	KX226384.1		
DogKHRovieng-12	Cambodia	Dog	I	B	KY548505.1		
DogKHRovieng-2	Cambodia	Dog	II	A(i)[A_1454]	KX226375.1		
DogKHRovieng-3	Cambodia	Dog	V	B	KX226376.1		
DogKHRovieng-4	Cambodia	Dog	I	B	KX226377.1		
DogKHRovieng-5	Cambodia	Dog	I and V	B	KX226378.1		
DogKHRovieng-6	Cambodia	Dog	I and V	B	KX226379.1		
DogKHRovieng-7	Cambodia	Dog	I	B	KX226380.1		
DogKHRovieng-8	Cambodia	Dog	IV and V	B	KX226381.1		
DogKHRovieng-9	Cambodia	Dog	V	B	KX226382.1		
HumKHRovieng-1	Cambodia	Human	I and II	A	KX226367.1		
HumKHRovieng-2	Cambodia	Human	II	A(i)[A_1454]	KX226368.1		
HumKHRovieng-3	Cambodia	Human	II and III	A	KX226369.1		
HumKHRovieng-4	Cambodia	Human	II	A	KX226370.1		
HumKHRovieng-5	Cambodia	Human	I and II	A	KX226371.1		
HumKHRovieng-6	Cambodia	Human	II	A	KX226372.1		
HumKHRovieng-7	Cambodia	Human	II	A	KX226373.1		
MA3	Thailand	Human	-	A	KY081224.1	Thanchomnang et al. [7]	The two *S*. *stercoralis* types from this study represent 18 worms with the A genotype and the *cox1* sequences KY081224 to KY081232, and KY081234 to KY081242.
UD33	Thailand	Human	-	S	KY081233.1		
UD45	Thailand	Human	-	A	KY081235.1		
1AStr1	Central African Republic (CAR)	Human	-	K	LC085504.1	Hasegawa et al. [26]	While a HVR-IV sequence is available for specimen 2AStr1, it was excluded from the genotyping scheme due to the presence of several ambiguous (N) bases. Specimen D448Str1 has a truncated HVR-IV sequence from *S*. *fuelleborni* which could represent either genotypes N or K. While a HVR-IV sequence is available for specimen Ch28Str1, it was excluded from the typing scheme due to the presence of several ambiguous (N) bases.
3AStr1	Central African Republic (CAR)	Human	-	M	-		
3EStr6	Central African Republic (CAR)	Human	-	A(ii)[G_1454]	LC085500.1		
4AStr4	Central African Republic (CAR)	Human	-	A(ii)[G_1454]	LC085498.1		
Ch28Str2	Central African Republic (CAR)	Chimpanzee	-	P	LC085507.1		
D430Str2	Central African Republic (CAR)	Chimpanzee	-	N	-		
D437Str2	Central African Republic (CAR)	Chimpanzee	-	K	-		
D450Str1	Central African Republic (CAR)	Chimpanzee	-	K	LC085511.1 ^γ^		
D450Str2	Central African Republic (CAR)	Chimpanzee	-	K	-		
D450Str3	Central African Republic (CAR)	Chimpanzee	-	K	LC085512.1 ^γ^		
D450Str4	Central African Republic (CAR)	Chimpanzee	-	K	LC085513.1 ^γ^		
Gor46Str1	Central African Republic (CAR)	Gorilla	-	L	LC085508.1		
Gor46Str2	Central African Republic (CAR)	Gorilla	-	L	LC085509.1		
3EStr3	Central African Republic (CAR)	Human	-	A(i)[A_1454]	-		
2AStr1	Central African Republic (CAR)	Human	-	LC085485.1	LC085502.1		
D448Str1	Central African Republic (CAR)	Chimpanzee	-	N or K	-		
Ch28Str1	Central African Republic (CAR)	Chimpanzee	-	LC085487.1	-		
SsLC17	Laos	Human	-	A	KU962139.1	Laymanivong et al. [18].	These genotypes represent 40 adult male worms each possessing the A genotype, and possessing GB *cox1* accessions KU962139 to KU962178.
SsLN1	Laos	Human	-	A	KU962157.1		
SsLS2	Laos	Human	-	A	KU962166.1		
SsLS27	Laos	Human	-	A	KU962168.1		
C51Sf	Gabon	Gorilla	XII	-	-	Makouloutou et al. [28]	NC
C139Sf	Gabon	Chimpanzee	XII	-	-		
NA—KF926658.1	Cambodia	Human	I	-	-	Schär et al. [17]	These genotypes represent 269 worms, collected from 29 individual humans.
NA—KF926659.1	Cambodia	Human	II	-	-		
NA—KF926660.1	Cambodia	Human	III	-	-		
NA—KF926662.1	Cambodia	Human	-	A(i)[A_1454]	-		
NA—KF926661.1	Cambodia	Human	-	A(ii)[G_1454]	-		
NA—AB677955.1	Japan	Japanese macaque	XIV	-	-	Giang, et al. 2017 [29]	NC
ChimpGabon-A	Gabon	Chimpanzee	-	P	-	Hasegawa et al. [16]	NC
ChimpTanzEM	Tanzania	Chimpanzee	-	K	-		
HumTanz-1	Tanzania	Human	-	A	AB526297.1		
YelBaboonTanz-1	Tanzania	Baboon	-	Q	AB526306.1		
ChimpTanzCE-6	Tanzania	Chimpanzee	-	K	-		
ChimpTanzCE-4	Tanzania	Chimpanzee	-	K	-		
GorillaGabon-D	Gabon	Gorilla	-	P	-		
ChimpTanzCE-2	Tanzania	Chimpanzee	-	A	AB526305.1		
NA—AB453317.1	Japan	Japanese macaque	XIV	S	-	Hasegawa et al. [15]	NC
NA—AB453320.1	Tanzania	Human	XII	K	-		
NA—AB453321.1	Gabon	Chimpanzee	XII	R	-		
NA—AB453322.1	Gabon	Gorilla	XIII	O	-		
NA—AB453318 .1	Japan	Japanese macaque	XIV	S	-		
NA—AB453319 .1	Japan	Japanese macaque	XIV	S	-		
JPN (Shoudoshima)	Japan	Japanese macaque	XIV	S	-	Sato et al. [30]	NC

^α^ Represents a cryptic *Strongyloides* sp. that may be present in this specimen due to coprophagy.

^β^ These *cox1* sequences were not shown in Fig 2 because they incompletely overlap with the 217 bp amplicon generated in this study. These sequences were identical to AJ558163.1 however (shown in Fig 2, a US dog) for the overlapping region of the sequence.

^γ^ These *cox1* sequences are not shown in Fig 2 because they incompletely overlap with the 217 bp fragment of *cox*1 generated in this study.

NA–no strain/isolate name assigned so a GB Accession was used instead to represent this specimen.

Note: Isolates/strains with no 18S data are not included in this table but are represented in Fig 2 and S1 Fig.

A dash (-) indicates that no information is available for this region/marker. NC means “No Comments”.

Schär et al. [17], expanded on Hasegawa’s work [15, 16] by sequencing the HVR-I and HVR-IV regions from 269 *S*. *stercoralis* larvae cultured from 29 humans from Cambodia. Based on nucleotide variations at positions 176 to 179, in combination with a SNP at position 1454 (relative to reference sequence, GB: AF279916), a genotyping scheme describing three separate HVR I haplotypes (I-III) was proposed, along with two distinct genotypes of HVR-IV (A 1454 and G 1454), described in Table 3 in comparison to the typing scheme used here. Laymanivong et al. [18] studied fragments of the 18S rRNA and *cox1* for forty male *S*. *stercoralis* collected from humans in Laos. A single 18S rRNA haplotype (HVR-IV haplotype A) was found in this group alongside twenty-four new haplotypes of *cox1*. Hasegawa et al. [26] sequenced HVR-IV and *cox1* from *Strongyloides* spp. collected in the Central African Republic from the stool of humans, gorillas and chimpanzees and identified several distinct sequence types belonging to *S*. *fuelleborni*.

Thanchomnang et al. [7, 22] described *cox1* haplotypes of multiple *S*. *stercoralis* and one *S*. *f*. *fuelleborni* from humans in Thailand [7] and *S*. *fuelleborni* from long-tailed macaques in Thailand and Laos [22]. Frias et al. [27] generated *cox1* sequences from *S*. *fuelleborni* and other cryptic *Strongyloides* spp. infecting primates in Malaysia, including the Bornean slow loris (*Nycticebus borneanus*). Using the Illumina-based methodology described here, we previously identified *Strongyloides* spp. sequences in dogs and humans from Australia, including a cryptic *cox1* sequence which clustered between *S*. *stercoralis* and *S*. *fuelleborni* (Fig 2, dog with black star), and another that clustered nearest to the *Strongyloides* sp. “loris” group (Fig 2, dog with black circle) identified by Frias et al. [27]. Makouloutou et al. [28] generated *S*. *fuelleborni* sequences from Western lowland gorillas (*Gorilla gorilla*) and chimpanzees (*Pan troglodytes*) whilst performing a study on *Oesophagostomum* sp. in Gabon (Table 5). Giang et al. (2017) [29] used GenBank sequences generated from Japanese Macaques (*Macaca fuscata*) living at high altitudes to compare to *Strongyloides ransomi* from Vietnamese pigs. In the first description of *Strongyloides robustus*, Sato et al. [30] refer to a reference sequence from *S*. *f*. *fuelleborni* obtained from a Japanese Macaque (GB: AB272235.1), though provide few additional details. Basso et al. [31] described infections in three domestic dogs from Switzerland, identifying each dog to be infected with *S*. *stercoralis* of the A lineage.

When data from these studies were collated and visualized, several trends became apparent. As part of these visualizations we assigned clusters to different colors, corresponding to the relationships observed (Fig 2, note the different branch colors). Firstly, we note clustering of *S*. *fuelleborni cox1* sequences by geography, and possibly by host preference. In a human specimen from India, we detected a *cox1* sequence that clustered in the magenta clade along with sequences from macaques and humans originating from mainland SE Asia. The *cox1* sequences from Japanese macaques (light blue branches) clustered closely with those from mainland SE Asian long-tailed macaques and humans (magenta branches), yet these Japanese types were sufficiently distinct to form a separate group (Fig 2). Sequences within the cluster highlighted in magenta (mainland SE Asia and one from India) were associated 18S HVR-IV haplotype S (Fig 1, Table 3). Four sequences from Japanese macaques also possessed HVR-IV haplotype S, yet unfortunately, none of these specimens had a *cox1* sequence associated with them. However, given that both *S*. *f*. *fuelleborni* groups (from mainland SE Asia and Japan) share HVR-IV haplotype S, we propose that the Japanese macaques lacking a *cox1* sequence probably belong to the light blue cluster, or possibly the magenta cluster (Fig 3). We also note that HVR-I haplotype XIV is seemingly specific to Japanese macaques though only five sequences representing this group were available in GenBank. Unfortunately, we found no information for 18S HVR-I from *S*. *f*. *fuelleborni* infecting SE Asian long-tailed macaques (Fig 3).

The *S*. *f*. *fuelleborni cox1* sequences within the pink cluster all originate from Tanzania, and include specimens from chimpanzees (*Pan troglodytes*), a human and a yellow baboon (*Papio cynocephalus*) (Fig 2). Only one *cox1* sequence could be assigned to an 18S haplotype, that being HVR-IV haplotype Q identified in a baboon (Fig 3), which represents the only example of this HVR-IV haplotype. The gray cluster includes *S*. *f*. *fuelleborni cox1* sequences obtained from gorillas, humans and chimpanzees from Gabon and the Central African Republic (i.e. central Africa). We observed 18S HVR-IV hapltypes K, L and P in association with *cox1* sequences from this gray cluster, each derived from *S*. *f*. *fuelleborni* that infect humans and African great apes. A single *S*. *fuelleborni cox1* sequence found in an Australian specimen (Human 333_Au), is most similar to sequences from the gray cluster (central Africa) but seems sufficiently different to be considered distinct (Fig 2, human with miniaturized Australia). This Australian *S*. *fuelleborni cox1* sequence was found in association with 18S HVR-I haplotype XII (Fig 1). Unfortunately, no 18S data are available that correspond to *cox1* sequences in the Malaysian *S*. *f*. *fuelleborni* cluster (Fig 2, blue), which includes *S*. *f*. *fuelleborni* detected in orangutans (*Pongo pygmaeus*), proboscis monkeys (*Nasalis larvatus*), silvered leaf monkeys (or silvery lutung, *Trachypithecus cristatus*) and long-tailed macaques [27]. HVR-I haplotype XII found in Australia also seems widely distributed amongst African *S*. *f*. *fuelleborni* types, aside from one sequence identified in gorilla feces collected in Gabon that was assigned to haplotype XIII, found in association with HVR-IV haplotype O (Fig 3). *Strongyloides* spp. *cox1* sequences from lorises form their own distinct cluster (Fig 2, cyan branches).

Nagayasu et al. [20] examined *cox1* sequences and the 18S HVR-I region though followed a different scheme to the one described here, which considered the number of repeated T bases in this 18S region (Fig 1, green bars between positions 90 and 99 for HVR-I). That scheme differentiated between genotypes possessing 4 or 5 repeated T bases where 18S HVR-I haplotypes I, V and VI are of the “4T” type while HVR-I haplotypes II, III are of the “5T” type. Nagayasu et al. [20] also considered variants at base 458 which is either an “A” or “T” (Fig 1, corresponding to position 373 of HVR-I). According to that scheme, *S*. *stercoralis* genotypes I, II, IV, V (and now XI found in this study) possess an “A” at this position while only HVR-I haplotypes III and VI possess a “T” here. Nagayasu et al. [20] tested almost 600 worms, and while it is difficult to compare these results directly to those of the present scheme due to fundamental differences in data presentation, we can confirm that the “5T” types and the “T 458” types are the most common globally. Indeed, we demonstrate that genotype II + A, is by far the most abundant global genotype of *S*. *stercoralis* (Fig 3), which corresponds to the “5T + T 458”, type defined by Nagayasu et al. [20]. Nagayasu et al. [20] also confirmed the existence of two distinct lineages of *S*. *stercoralis*, these being type A and type B. The present study, like the study by Jaleta et al. [19], also supports the existence of these two lineages, where the type A and B lineages reported by Nagayasu et al. [20] conveniently correspond to *S*. *stercoralis* 18S HVR-IV haplotypes A and B, respectively. Canine cases of strongyloidiasis have thus far been attributed only to the zoonotic lineage of *S*. *stercoralis* (lineage A) in the Americas (the USA and Brazil) and Europe (Italy and Switzerland), though due to limited sampling, we cannot discard the possibility that the canine-specific lineage (lineage B) also exists outside of SE Asia.

Given that *cox1* sequences assigned to the green/purple cluster have never occurred in association with HVR-IV haplotype A and that *cox1* sequences of the red cluster have never occurred with HVR-IV haplotype B based on data collated from hundreds of specimens (Fig 2 and Fig 3), we suggest that interbreeding between these two lineages does not occur. This is despite the fact we clearly showed that co-infections with these two lineages do occur in dogs suggesting the distribution of these genotypes overlap, providing an opportunity for sexual crosses between them to take place if it were possible (see Beknazarova et al. [21], specimen “Dog 6”). This inability to sexually reproduce, if biologically proven, would support the assignment of *S*. *stercoralis* lineage B to *Strongyloides canis*, as suggested by Jaleta et al. [19].

A limitation of this study was the incapacity in many cases to actively collect samples from the countries of origin, necessitating the use of de-identified post-diagnostic samples for the analysis. The Australian, Cambodian, US, Guatemalan and Brazilian human and dog samples, the Italian dog samples and the Gambian Baboon sample were all collected in those countries of origin from subjects believed to have been infected locally. The human samples from other regions of Asia and Africa were obtained from refugees screened upon arrival at a clinic in Italy. While it is likely that infection was acquired in the country of origin for these latter samples, many such subjects have traversed multiple countries during their movement to Europe and thus the country of infection origin assigned remains presumptive. An improved study including prospective sampling of patients with selection of subjects to ensure that the country of most likely infection origin is indicated and more specific geographic data (village or city and state/province) is indicated to confirm the results of this study in the future.

In addition to the novel *Strongyloides* spp. 18S genotypes we detected previously [21], we report three novel haplotypes here, including HVR-IV haplotype J assigned to *S*. *stercoralis*, detected in a human specimen from the USA. We also discovered HVR-I haplotype XI of *S*. *stercoralis*, detected in humans from Australia and the Côte d'Ivoire. Additionally, a novel HVR-IV haplotype assigned to *S*. *fuelleborni* (haplotype T) was detected in a human specimen from Australia. Some genotypes we previously identified in Australian dogs were described as “cryptic” because they could not be confidently assigned to a species [21]. For these types, doubt was cast on whether they came from *Strongyloides* spp. truly parasitizing domestic dogs or were spuriously present in dog fecal specimens due to coprophagy or consumption of reptiles by dogs, because some haplotypes resembled sequences from reptile-infecting *Strongyloides*. These sequences were added to the genotyping scheme nonetheless (Fig 1), which some might argue is not an ideal solution. However, when it is considered that a sequence assigned to *S*. *f*. *fuelleborni* (GB: AJ417030.1) is identical to multiple *S*. *stercoralis* sequences available in GenBank, and a sequence assigned to *S*. *f*. *kellyi* (GB: AJ417029.1) is identical to sequences of *S*. *venezuelensis*, *S*. *vituli* and *S*. *cebus* and two of five sequences from GenBank assigned to *S*. *papillosus*, the inclusion of cryptic sequences is well justified. While these two sequences (GB: AJ417029.1 and AJ417030.1) represent a taxonomic/morphometric challenge to be addressed at a later time, they demonstrate that typing schemes avoiding species assignments can be extremely helpful in the context of genotype discovery and to facilitate straightforward comparisons between types. It is also very convenient to include the two causative agents of human strongyloidiasis in the same scheme. Additionally, it does not matter if genotype designations are assigned to sequences from cryptic *Strongyloides* spp. as they are discovered, because a species name can always be assigned to that genotype at a later time, accompanied by a detailed species description and appropriate morphometric analysis.

It is worthy to note that three human fecal specimens from Australia (Human 563_Au, Human 368_16_Au, Human 333_Au) contained *S*. *fuelleborni* sequences, and these represent the first detections of this species in Australia. Strongyloidiasis is recognized as an ongoing public health challenge in Northern and Central Australia, with high rates of infection in Australian Aboriginal communities [32]. The presence of *S*. *fuelleborni* human infections in Australia has implications for both the diagnosis and clinical management of the disease in Australia. The presence of *S*. *stercoralis* in Australia has prompted the development and/or implementation of molecular techniques for screening at-risk populations [23, 33, 34]. However, examination of the primers and probes from those studies [23, 33, 34] suggest a potential for cross-reactivity with *S*. *fuelleborni* DNA in some cases, given that one or more mismatches in priming sites and probes can still result in amplification products, though with a reduction in quantification accuracy resulting from reduced amplification efficiency [35, 36].

Robertson et al. [33] described two *S*. *stercoralis* real-time PCR positive samples from Queensland which generated an extra band on Single-Strand Conformation Polymorphism (SSCP) analysis when compared to other *S*. *stercoralis* from that region. We suggest this might represent detection of DNA from this Australian *S*. *fuelleborni*, previously not known to exist, particularly given that the primers used in that analysis would almost certainly amplify a product for *S*. *fuelleborni* [33]. Further evaluation of this Australian *S*. *fuelleborni* species is strongly indicated, including determination of its geographic distribution across the continent of Australia in the absence of native non-human primates, the clinical effects on infected people, determination of any zoonotic reservoirs of disease, reaction in standard diagnostic tests such as *Strongyloides* serologic assays and PCRs used in Australia, phylogenetic comparison to *S*. *f*. *kellyi* in Papua New Guinea and comprehensive morphological descriptions of all life stages of this strain.

A number of sequences corresponding to *Necator americanus* from human samples in Cambodia (Human 2_Ca), Laos (Human 1_La) and northern Australia (Human 333_Au and Human 378_Au) were detected here. Furthermore, two sequences similar to those from the human-infecting parasite *Oesophogostomum bifurcum* were identified from one Australian fecal sample (Human 434_Au). Notably, *O*. *bifurcum* is a parasite of non-human primates that may occasionally infect humans, particularly within some limited geographic foci in West Africa [37]. This sample, from the state of Victoria, was the only sample analyzed from outside of the tropical north of the continent. A large African migrant diaspora has settled in Victoria and we speculate that this finding may represent a long-standing infection originally acquired in Africa prior to immigration to Australia and incidentally discovered in the process of this study. In two instances where strongylid DNA was detected, *Strongyloides* 18S data was generated, but only strongylid *cox1* sequences (*N*. *americanus* in Human 1_La, and *O*. *bifurcum* in Human 434_Au, Fig 4) were obtained. Similarly, specimen Human 2_Ca generated a *S*. *stercoralis* 18S HVR-I amplicon, but sequences from both *N*. *americanus* and *S*. *stercoralis* were identified in the *cox1* amplicon. We suggest that in the latter two cases, the intensity of shedding was higher for the strongylids, meaning that amplification of the strongylid *cox1* DNA was favored over *S*. *stercoralis* in the *cox1* PCR. The 18S primers were designed to be specific for members of the genus *Strongyloides*, meaning that there was no competition for amplification of the 18S with DNA from other species, resulting in the amplification of only *Strongyloides* DNA at this locus.

Our Illumina-based methodology has several advantages over previous methods, including the ability to detect multiple haplotypes in DNA extracted directly from stool without having to culture *Strongyloides* spp. larvae. It also allows investigators to detect infections and co-infections involving multiple gastrointestinal nematode genera (Fig 4). However, we also recognize its disadvantages over previous approaches. Firstly, as discussed above, when co-infections involving a *Strongyloides* sp. and a strongylid were detected, Illumina sequencing sometimes only returned *cox1* data for the strongylid. Sequencing *cox1* amplicons at a greater depth by including fewer specimens in Illumina libraries when multiplexing, or by using an Illumina sequencing kit with a higher number of amplification cycles might overcome this. A further disadvantage of our protocol when it is applied directly to stool is that when two haplotypes are detected for two or all loci examined here, it is impossible to determine the underlying genotypes of individual worms, limiting our ability to identify genetically isolated populations in a mixed infection. Under the conditions described, the assay only returned a 63% to 80% success rate (Table 1). This meant that in a significant number of cases, the genotyping data was incomplete. It is likely that this is due to the concentration of *Strongyloides* DNA present in the samples being below that detectable for the given targets. This problem might be remedied in the future by undertaking a simple larval recovery method, such as Baermann concentration, followed by bulk extraction of all larvae recovered from each individual specimen, similar to the approach used in the recently described veterinary “nemabiome” assay [38].

Another weakness of this approach is the fact that long amplicons are difficult to analyze by nature of NEBNext Ultra DNA kit utilized here, which results in coverage biased towards the ends of amplicons due to a lack of tagmentation. Indeed, the HVR-I amplicon was the longest of the loci (~450 bp including the primer sequence), and was the most challenging to analyze because low coverage in the middle of these amplicons meant that merging was not possible for many read pairs. However, while slightly more challenging, this was not a major issue here as the middle of the HVR-I amplicon is identical for all haplotypes (Fig 1) and the pairing information could still be used to construct haplotypes. Regardless, a solution could involve using the Illumina MiSeq V3 reagent kit which results in 300 bp paired-end reads as opposed to the 250 bp reads generated here using the Nano Kit v2.

The improved ease and utility of genotyping directly from fecal samples provided by this assay opens the capacity to undertake largescale genotyping studies of *Strongyloides* species for epidemiologic studies and pathogen discovery. The capacity for cost-effective and relatively rapid largescale testing afforded by this assay will assist in determining overall genetic diversity of the genus. Further applications of this method include better understanding of the transmission dynamics of strongyloidiasis and the association of human infections to animal reservoirs in specific regions. Application of this methodology may assist in determining the geographic source of infections, or source of infection in travel-associated cases. Finally, the detection of new genotypes and assessment of the global diversity of genotypes in these clinically important species affords the opportunity to determine the individual clinical effects and host range of each genotype.

### Conclusions

The *Strongyloides* spp. genotyping assay described provides important advantages over previously described methods. For instance, we demonstrated its capacity to detect and differentiate DNA from different *Strongyloides* spp. genotypes and also multiple strongylids in DNA extracted directly from stool. We also show that the data generated using this assay remains compatible with data generated using earlier approaches based on Sanger sequencing, facilitating direct comparison of new data generated using this assay with published data. As this analysis represents more than 1,000 sequences collected from a diverse range of hosts and world locations, it provides a truly global understanding of the population genetic structure of the *Strongyloides* spp. infecting humans, non-human primates and domestic dogs.

## Supporting information

S1 Fig Cluster dendrogram of *cox1* sequences with GB accessions numbers provided. This file serves as an aid for Fig 1 as it shows an identical dendrogram to Fig 1, yet also provides all GB accession numbers for the sequences included, along with the country of origin and host species. The file allows zooming without loss of resolution, and is also searchable for readers who wish to search the position of specific accession numbers using the ‘Find’ function in Adobe Acrobat PDF reader or another preferred PDF reader.
(PDF)

S1 File Excel spreadsheet containing BioSample numbers and GenBank accession numbers. (XLSX)

S2 File Fasta file of sequences used to construct Fig 2. (FASTA)

S3 File Fasta file of sequences used to construct Fig 4. (FASTA)

## References

[1] Olsen A, van Lieshout L, Marti H, Polderman T, Polman K, Steinmann P, et al Strongyloidiasis—the most neglected of the neglected tropical diseases? Trans R Soc Trop Med Hyg. 2009;103(10):967–72. Epub 2009/03/31. 10.1016/j.trstmh.2009.02.013 . 19328508

[2] Speare R. Identification of species of *Strongyloides* Strongyloidiasis a major roundworm infection of man. Grove D.I., ed. ed. London: Taylor and Francis; 1989 p. 11–84.

[3] Bisoffi Z, Buonfrate D, Montresor A, Requena-Mendez A, Munoz J, Krolewiecki AJ, et al *Strongyloides stercoralis*: a plea for action. PLoS Negl Trop Dis. 2013;7(5):e2214 Epub 2013/05/16. 10.1371/journal.pntd.0002214 23675546 PMC3649953

[4] Strkolcova G, Goldova M, Bockova E, Mojzisova J. The roundworm *Strongyloides stercoralis* in children, dogs, and soil inside and outside a segregated settlement in Eastern Slovakia: frequent but hardly detectable parasite. Parasitol Res. 2017;116(3):891–900. Epub 2017/01/12. 10.1007/s00436-016-5362-1 . 28074315

[5] Beknazarova M, Whiley H, Ross K. Strongyloidiasis: A Disease of Socioeconomic Disadvantage. Int J Environ Res Public Health. 2016;13(5). Epub 2016/05/24. 10.3390/ijerph13050517 27213420 PMC4881142

[6] Agarwala R, Wasielewski J, Biman B. Pulmonary strongyloidiasis following renal transplantation without travel to an endemic area. Oxf Med Case Reports. 2014;2014(4):83–5. Epub 2015/05/20. 10.1093/omcr/omu032 25988037 PMC4399508

[7] Thanchomnang T, Intapan PM, Sanpool O, Rodpai R, Tourtip S, Yahom S, et al First molecular identification and genetic diversity of *Strongyloides stercoralis* and *Strongyloides fuelleborni* in human communities having contact with long-tailed macaques in Thailand. Parasitol Res. 2017;116(7):1917–23. Epub 2017/05/14. 10.1007/s00436-017-5469-z . 28500375

[8] Pampiglione S, Ricciardi ML. Experimental infestation with human strain *Strongyloides fulleborni* in man. Lancet. 1972;1(7752):663–5. Epub 1972/03/25. 10.1016/s0140-6736(72)90464-3 . 4125165

[9] Nutman TB. Human infection with *Strongyloides stercoralis* and other related *Strongyloides* species. Parasitology. 2017;144(3):263–73. Epub 2016/05/18. 10.1017/S0031182016000834 27181117 PMC5563389

[10] Hira P, Patel B. Human strongyloidiasis due to the primate species *Strongyloides fuelleborni*. Tropical and geographical medicine. 1980;32(1):23–9. 7394891

[11] Pampiglione S, Ricciardi ML. The presence of *Strongyloides fulleborni* von Linstow, 1905, in man in Central and East Africa. Parassitologia. 1971;13(1):257–69. Epub 1971/04/01. . 5152940

[12] Ashford RW, Barnish G, Viney ME. *Strongyloides fuelleborni kellyi*: infection and disease in Papua New Guinea. Parasitol Today. 1992;8(9):314–8. Epub 1992/09/01. 10.1016/0169-4758(92)90106-c . 15463651

[13] Viney M, Ashford R, Barnish G. A taxonomic study of *Strongyloides* Grassi, 1879 (Nematoda) with special reference to *Strongyloides fuelleborni* von Linstow, 1905 in man in Papua New Guinea and the description of a new subspecies. Systematic parasitology. 1991;18:95–109.

[14] Dorris M, Viney ME, Blaxter ML. Molecular phylogenetic analysis of the genus *Strongyloides* and related nematodes. Int J Parasitol. 2002;32(12):1507–17. Epub 2002/10/24. 10.1016/s0020-7519(02)00156-x . 12392916

[15] Hasegawa H, Hayashida S, Ikeda Y, Sato H. Hyper-variable regions in 18S rDNA of *Strongyloides* spp. as markers for species-specific diagnosis. Parasitol Res. 2009;104(4):869–74. Epub 2008/12/04. 10.1007/s00436-008-1269-9 . 19050926

[16] Hasegawa H, Sato H, Fujita S, Nguema PP, Nobusue K, Miyagi K, et al Molecular identification of the causative agent of human strongyloidiasis acquired in Tanzania: dispersal and diversity of *Strongyloides* spp. and their hosts. Parasitol Int. 2010;59(3):407–13. Epub 2010/07/14. 10.1016/j.parint.2010.05.007 . 20621633

[17] Schar F, Guo L, Streit A, Khieu V, Muth S, Marti H, et al *Strongyloides stercoralis* genotypes in humans in Cambodia. Parasitol Int. 2014;63(3):533–6. Epub 2014/02/18. 10.1016/j.parint.2014.01.010 . 24530857

[18] Laymanivong S, Hangvanthong B, Insisiengmay B, Vanisaveth V, Laxachack P, Jongthawin J, et al First molecular identification and report of genetic diversity of *Strongyloides stercoralis*, a current major soil-transmitted helminth in humans from Lao People's Democratic Republic. Parasitol Res. 2016;115(8):2973–80. Epub 2016/04/17. 10.1007/s00436-016-5052-z . 27083185

[19] Jaleta TG, Zhou S, Bemm FM, Schar F, Khieu V, Muth S, et al Different but overlapping populations of *Strongyloides stercoralis* in dogs and humans-Dogs as a possible source for zoonotic strongyloidiasis. PLoS Negl Trop Dis. 2017;11(8):e0005752 Epub 2017/08/10. 10.1371/journal.pntd.0005752 28793306 PMC5565190

[20] Nagayasu E, Aung M, Hortiwakul T, Hino A, Tanaka T, Higashiarakawa M, et al A possible origin population of pathogenic intestinal nematodes, *Strongyloides stercoralis*, unveiled by molecular phylogeny. Sci Rep. 2017;7(1):4844 Epub 2017/07/09. 10.1038/s41598-017-05049-x 28687738 PMC5501853

[21] Beknazarova M, Barratt J, Bradbury R, Lane M, Whiley H, Ross K. Detection of classic and cryptic Strongyloides genotypes by deep amplicon sequencing: A preliminary survey of dog and human specimens collected from remote Australian communities. PLoS neglected tropical diseases. 2019; PLoS Negl Trop Dis 13(8): e0007241 10.1371/journal.pntd.0007241 31430282 PMC6716672

[22] Thanchomnang T, Intapan PM, Sanpool O, Rodpai R, Sadaow L, Phosuk I, et al First molecular identification of Strongyloides fuelleborni in long-tailed macaques in Thailand and Lao People's Democratic Republic reveals considerable genetic diversity. J Helminthol. 2018:1–8. Epub 2018/07/22. 10.1017/S0022149X18000512 . 30027858

[23] Verweij JJ, Canales M, Polman K, Ziem J, Brienen EA, Polderman AM, et al Molecular diagnosis of *Strongyloides stercoralis* in faecal samples using real-time PCR. Trans R Soc Trop Med Hyg. 2009;103(4):342–6. Epub 2009/02/07. 10.1016/j.trstmh.2008.12.001 . 19195671

[24] Koga K, Kasuya S, Khamboonruang C, Sukhavat K, Ieda M, Takatsuka N, et al A modified agar plate method for detection of *Strongyloides stercoralis*. Am J Trop Med Hyg. 1991;45(4):518–21. Epub 1991/10/01. 10.4269/ajtmh.1991.45.518 . 1951861

[25] Iatta R, Buonfrate D, Paradies P, Cavalera MA, Capogna A, Iarussi F, et al Occurrence, diagnosis and follow-up of canine strongyloidiosis in naturally infected shelter dogs. Parasitology. 2019;146(2):246–52. Epub 2018/07/31. 10.1017/S0031182018001312 . 30058514

[26] Hasegawa H, Kalousova B, McLennan MR, Modry D, Profousova-Psenkova I, Shutt-Phillips KA, et al *Strongyloides* infections of humans and great apes in Dzanga-Sangha Protected Areas, Central African Republic and in degraded forest fragments in Bulindi, Uganda. Parasitol Int. 2016;65(5 Pt A):367–70. Epub 2016/05/18. 10.1016/j.parint.2016.05.004 . 27180094

[27] Frias L, Stark DJ, Lynn MS, Nathan SK, Goossens B, Okamoto M, et al Lurking in the dark: Cryptic *Strongyloides* in a Bornean slow loris. Int J Parasitol Parasites Wildl. 2018;7(2):141–6. Epub 2018/07/11. 10.1016/j.ijppaw.2018.03.003 29988792 PMC6031959

[28] Makouloutou P, Mbehang Nguema P, Fujita S, Takenoshita Y, Hasegawa H, Yanagida T, et al Prevalence and genetic diversity of *Oesophagostomum stephanostomum* in wild lowland gorillas at Moukalaba-Doudou National Park, Gabon. Helminthologia. 2014;51(2):83–93.

[29] Giang NT, Hoan TD, Huyen NTT, Lan NTK, Doanh PN. Morphological and molecular characterisation of Strongyloides ransomi (Nematoda: Strongyloididae) collected from domestic pigs in Bac Giang province, Vietnam. *Tap Chi Sinh Hoc*. 2017;39(3):270–275.

[30] Sato H, Torii H, Une Y, Ooi HK. A new rhabditoid nematode species in Asian sciurids, distinct from *Strongyloides robustus* in North American sciurids. J Parasitol. 2007;93(6):1476–86. Epub 2008/03/05. 10.1645/GE-1106.1 . 18314696

[31] Basso W, Grandt LM, Magnenat AL, Gottstein B, Campos M. *Strongyloides stercoralis* infection in imported and local dogs in Switzerland: from clinics to molecular genetics. Parasitol Res. 2019;118(1):255–66. Epub 2018/12/16. 10.1007/s00436-018-6173-3 . 30552576

[32] Page W, Judd JA, Bradbury RS. The Unique Life Cycle of *Strongyloides stercoralis* and Implications for Public Health Action. Trop Med Infect Dis. 2018;3(2). Epub 2018/10/03. 10.3390/tropicalmed3020053 30274449 PMC6073624

[33] Robertson GJ, Koehler AV, Gasser RB, Watts M, Norton R, Bradbury RS. Application of PCR-Based Tools to Explore Strongyloides Infection in People in Parts of Northern Australia. Trop Med Infect Dis. 2017;2(4). Epub 2018/10/03. 10.3390/tropicalmed2040062 30270919 PMC6082066

[34] Watts MR, Kim R, Ahuja V, Robertson GJ, Sultana Y, Wehrhahn M, et al Comparison of loop-mediated isothermal amplification (LAMP) and real-time polymerase chain reaction (PCR) assays for the detection of Strongyloides in different specimen matrices. J Clin Microbiol. 2019 Epub 2019/02/08. 10.1128/JCM.01173-18 . 30728195 PMC6440779

[35] Ledeker BM, De Long SK. The effect of multiple primer-template mismatches on quantitative PCR accuracy and development of a multi-primer set assay for accurate quantification of pcrA gene sequence variants. J Microbiol Methods. 2013;94(3):224–31. Epub 2013/06/29. 10.1016/j.mimet.2013.06.013 . 23806694

[36] Stadhouders R, Pas SD, Anber J, Voermans J, Mes TH, Schutten M. The effect of primer-template mismatches on the detection and quantification of nucleic acids using the 5' nuclease assay. J Mol Diagn. 2010;12(1):109–17. Epub 2009/12/02. 10.2353/jmoldx.2010.090035 19948821 PMC2797725

[37] Polderman AM, Blotkamp J. Oesophagostomum infections in humans. Parasitol Today. 1995;11(12):451–6. Epub 1995/12/01. 10.1016/0169-4758(95)80058-1 . 15275382

[38] Avramenko RW, Redman EM, Lewis R, Bichuette MA, Palmeira BM, Yazwinski TA, et al The use of nemabiome metabarcoding to explore gastro-intestinal nematode species diversity and anthelmintic treatment effectiveness in beef calves. Int J Parasitol. 2017;47(13):893–902. Epub 2017/08/12. 10.1016/j.ijpara.2017.06.006 . 28797791

[39] Zhou S, Fu X, Pei P, Kucka M, Liu J, Tang L, et al Characterization of a non-sexual population of *Strongyloides stercoralis* with hybrid 18S rDNA haplotypes in Guangxi, Southern China. PLoS Negl Trop Dis. 13(5): e0007396 Epub 2019/05/06. 10.1371/journal.pntd.0007396 31059500 PMC6522072

