# Correction: A global genotyping survey of *Strongyloides stercoralis* and *Strongyloides fuelleborni* using deep amplicon sequencing

**DOI:** 10.1371/journal.pntd.0009538

**Published:** 2021-06-18

**Authors:** Joel L. N. Barratt, Meredith Lane, Emir Talundzic, Travis Richins, Gemma Robertson, Fabio Formenti, Bobbi Pritt, Guilherme Verocai, Joelma Nascimento de Souza, Neci Mato Soares, Rebecca Traub, Dora Buonfrate, Richard S. Bradbury

All errors found in this paper are due to six samples included in the paper incorrectly assigned as being from Queensland, Australia. The report of *Strongyloides fuelleborni* infections from Australia made in this paper was incorrect, as those samples in fact originated in Guinea-Bissau and Senegal.

There is an error in Table 4. Specimen Human 333_Au from Queensland (Australia) should be listed as Human 333_GuBi from Guinea-Bissau. Specimen Human 368_16_Au from Queensland (Australia) should be listed as Human 368_16_Se from Senegal. Specimen Human 378_Au from Queensland (Australia) should be listed as Human 378_Bo from Bolivia. Specimen Human 507_Au from Queensland (Australia) should be listed as Human 507_Ni from Nigeria. Specimen Human 524_Au from Queensland (Australia) should be listed as Human 524_Ni from Nigeria. Specimen Human 563_Au from Queensland (Australia) should be listed as Human 563_GuBi from Guinea-Bissau. The authors have provided a corrected Table 4 with the corrected specimens and locations in red.

**Table 4:** Human, primate and dog *Strongyloides* spp. specimens analyzed in this study and their genotype.

Specimen Name	Location	Host	HVR-I haplotype	HVR-IV haplotype	*Cox*1 Accessions	Helminth/s detected
Baboon 5_N8_Ga	Gambia	Papio papio	XII	-	-	*S*. *fuelleborni*
Dog 1101_It	Italy	Dog	VI	A	MN076424	*S*. *stercoralis*
Dog 1141_It	Italy	Dog	VI	-	MN076425	*S*. *stercoralis*
Dog 4_Ca	Cambodia	Dog	-	A	-	*S*. *stercoralis*
Dog 5_US_Cl_OH	Ohio (USA)	Dog	VI	-	-	*S*. *stercoralis*
Dog 7_Ca	Cambodia	Dog	V	B	-	*S*. *stercoralis*
Dog A4_US_GA	Georgia (USA)	Dog	VI	A	MN076426	*S*. *stercoralis*
Dog A7_US_GA	Georgia (USA)	Dog	VI	A	MN076427	*S*. *stercoralis*
Dog Bu_US_Cl_OH	Cleveland (USA)	Dog	VI	A	MN076428	*S*. *stercoralis*
Dog US_ATL_GA	Georgia (USA)	Dog	VI	-	MN076429	*S*. *stercoralis*
Dog US_GA1	Georgia (USA)	Dog	-	-	MN076430	*S*. *stercoralis*
Dog US_PA	Pennsylvania (USA)	Dog	I and VI	-	MN076431	*S*. *stercoralis*
Dog W2_US_GA	Georgia (USA)	Dog	VI	-	MN076432	*S*. *stercoralis*
Human 1_Ca	Cambodia	Human	-	A	MN076433	*S*. *stercoralis*
Human 1_La	Laos	Human	II	A	MN076459	*S*. *stercoralis*, *Necator americanus*
Human 10_An	Angola	Human	-	A	-	*S*. *stercoralis*
Human 100_Et	Ethiopia	Human	-	A	-	*S*. *stercoralis*
Human 1070_Se	Senegal	Human	-	A	-	*S*. *stercoralis*
Human 10VS_US_KY	Kentucky (USA)	Human	VI	A	-	*S*. *stercoralis*
Human 1207_Br	Bahia (Brazil)	Human	-	A	-	*S*. *stercoralis*
Human 14WC_US_LA	Louisiana (USA)	Human	II and VI	A and J	MN076434	*S*. *stercoralis*
Human 1540_Ni	Nigeria	Human	II	A	MN076451	*S*. *stercoralis*
Human 1598_Gu_Co	Guinea (Conakry)	Human	II	A	MN076452	*S*. *stercoralis*
Human 169_Et	Ethiopia	Human	I and II	A	MN076438	*S*. *stercoralis*
Human 2_Ca	Cambodia	Human	II	NA	MN076460	*S*. *stercoralis*, *N*. *americanus*
Human 21_17_La	Laos	Human	VI	-	MN076435	*S*. *stercoralis*
Human 229_Au	Queensland (Australia)	Human	I	-	MN076439	*S*. *stercoralis*
Human 238_Au	Queensland (Australia)	Human	II	A	MN076440	*S*. *stercoralis*
Human 25_17_La	Laos	Human	VI	A	-	*S*. *stercoralis*
Human 3_Ca	Cambodia	Human	II	A	-	*S*. *stercoralis*
Human 308_Au	Queensland (Australia)	Human	II	A	MN076441	*S*. *stercoralis*
Human333_Gubi	Guinea‐Bissau	Human	XII	-	MN076442, MN076461	*S*. *fuelleborni*, *N*. *americanus*
Human 349_7_Au	Queensland (Australia)	Human	II	A	MN076443	*S*. *stercoralis*
Human 352_Au	Queensland (Australia)	Human	-	A	-	*S*. *stercoralis*
Human 358_Au	Queensland (Australia)	Human	-	A	-	*S*. *stercoralis*
Human 360_Au	Queensland (Australia)	Human	II	-	-	*S*. *stercoralis*
Human 367_Au	Queensland (Australia)	Human	II	A	MN076444	*S*. *stercoralis*
Human368_16_Se	Senegal	Human	XII	M and T	-	*S*. *fuelleborni*
Human378_Bo	Bolivia	Human	III and XI	A	MN076445, MN076462	*S*. *stercoralis*, *N*. *americanus*
Human 395_Au	Queensland (Australia)	Human	II	A	-	*S*. *stercoralis*
Human 428_Au	Queensland (Australia)	Human	II	A	-	*S*. *stercoralis*
Human 434_Au	Victoria (Australia)	Human	II	-	MN076463, MN076464	*S*. *stercoralis*, *Oesophagostomum* sp.
Human 441_Au	Queensland (Australia)	Human	II	-	MN076446	*S*. *stercoralis*
Human507_Ni	Nigeria	Human	I	-	MN076447	*S*. *stercoralis*
Human 519_Au	Queensland (Australia)	Human	II	A	-	*S*. *stercoralis*
Human524_Ni	Nigeria	Human	I	A	MN076448	*S*. *stercoralis*
Human 528_Au	Queensland (Australia)	Human	II	A	-	*S*. *stercoralis*
Human 5325_In	India	Human	-	S	MN076453	*S*. *fuelleborni*
Human 5333_It	Italy	Human	I and III	A	MN076454	*S*. *stercoralis*
Human 5344_Br	Bahia (Brazil)	Human	I	A	MN076455	*S*. *stercoralis*
Human563_GuBi	Guinea‐Bissau	Human	XII	-	-	*S*. *fuelleborni*
Human 58_It	Italy	Human	III	A	MN076436	*S*. *stercoralis*
Human 588_Au	Queensland (Australia)	Human	II	-	MN076449	*S*. *stercoralis*
Human 877_IvCo	Ivory Coast	Human	XI	A	MN076450	*S*. *stercoralis*
Human 88_GuBi	Guinea-Bissau	Human	-	-	MN076437	*S*. *stercoralis*
Human 930_IvCo	Ivory Coast	Human	II	A	-	*S*. *stercoralis*
Human A3_Br	Bahia (Brazil)	Human	-	A	MN076456	*S*. *stercoralis*
Human A4_Br	Bahia (Brazil)	Human	I and III	A	-	*S*. *stercoralis*
Human A9_Br3	Bahia (Brazil)	Human	II	A	MN076457	*S*. *stercoralis*
Human Et_Au	Western Australia	Human	II	-	MN076458	*S*. *stercoralis*

NA: Specimen excluded due to contamination with our *Strongyloides ratti* control DNA

-: Sequence not obtained due to amplification and/or sequencing failure

Notes: GenBank Accession numbers for the HVR-I and HVR-IV sequences and BioSample numbers (for linking each specimen to its associated raw Illumina data) are provided in S1 File. Red text indicates specimens with amended details.

Fig 1 is incorrect due to the error in Table 4. The authors have provided a corrected version here.

**Fig 1:**
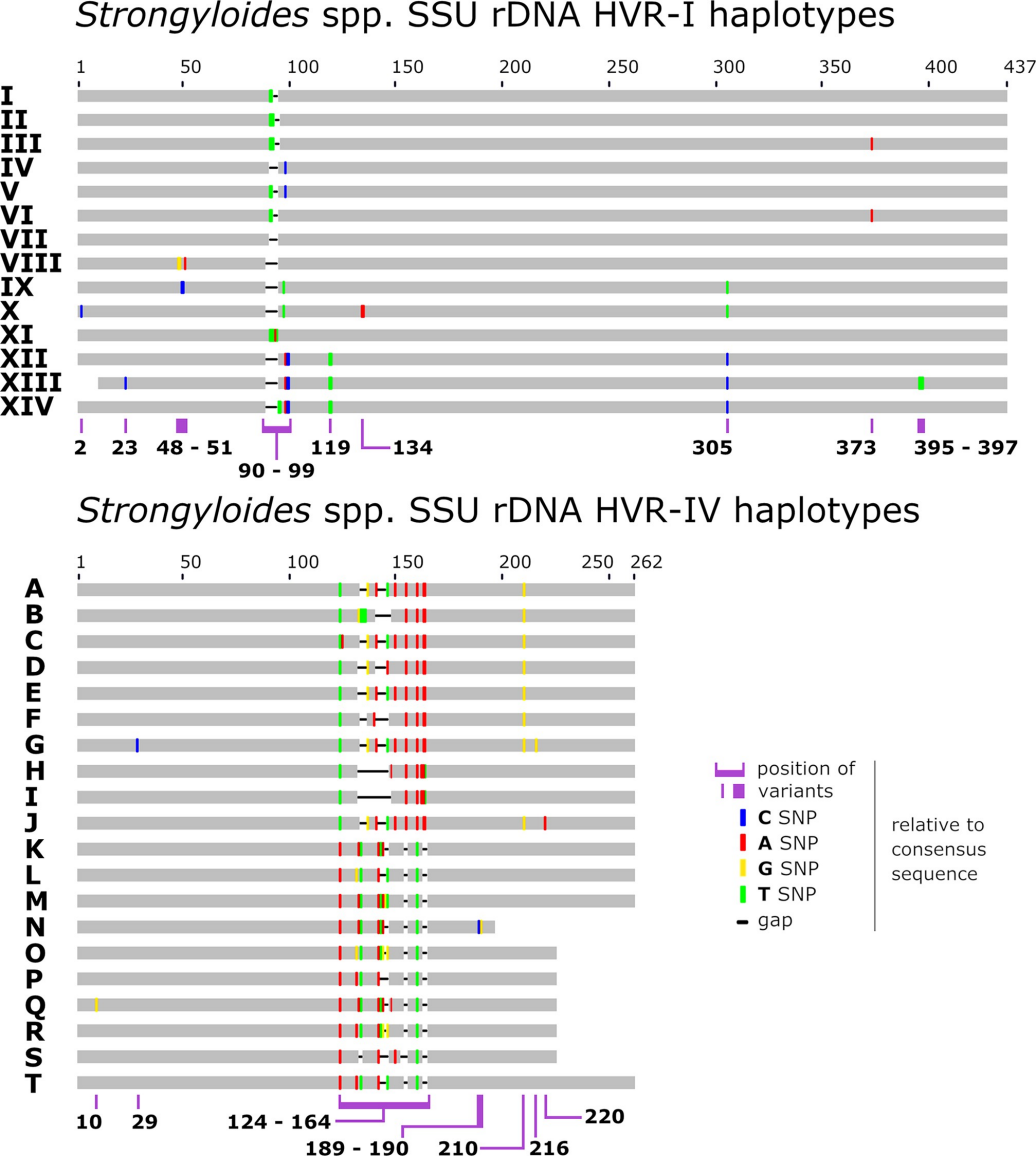
Schematic of the *Strongyloides* spp. genotyping scheme. A graphical representation of the *Strongyloides* spp. genotyping scheme described previously [21], expanded to include additional genotypes from *S*. *stercoralis* and *S*. *fuelleborni*. This scheme includes novel sequences identified by Barratt et al., and 18S sequences that were available in GenBank (GB), where the appropriate 18S HVR-I and/or HVR-IV regions were captured. Sequences from GB possessing Ns or ambiguous bases were excluded from the scheme. For additional details on the hosts in which these *Strongyloides* spp. haplotypes were detected refer to the Tables in Barratt et al.

Fig 2 is incorrect due to the error in Table 4. The authors have provided a corrected version here.

**Fig 2:**
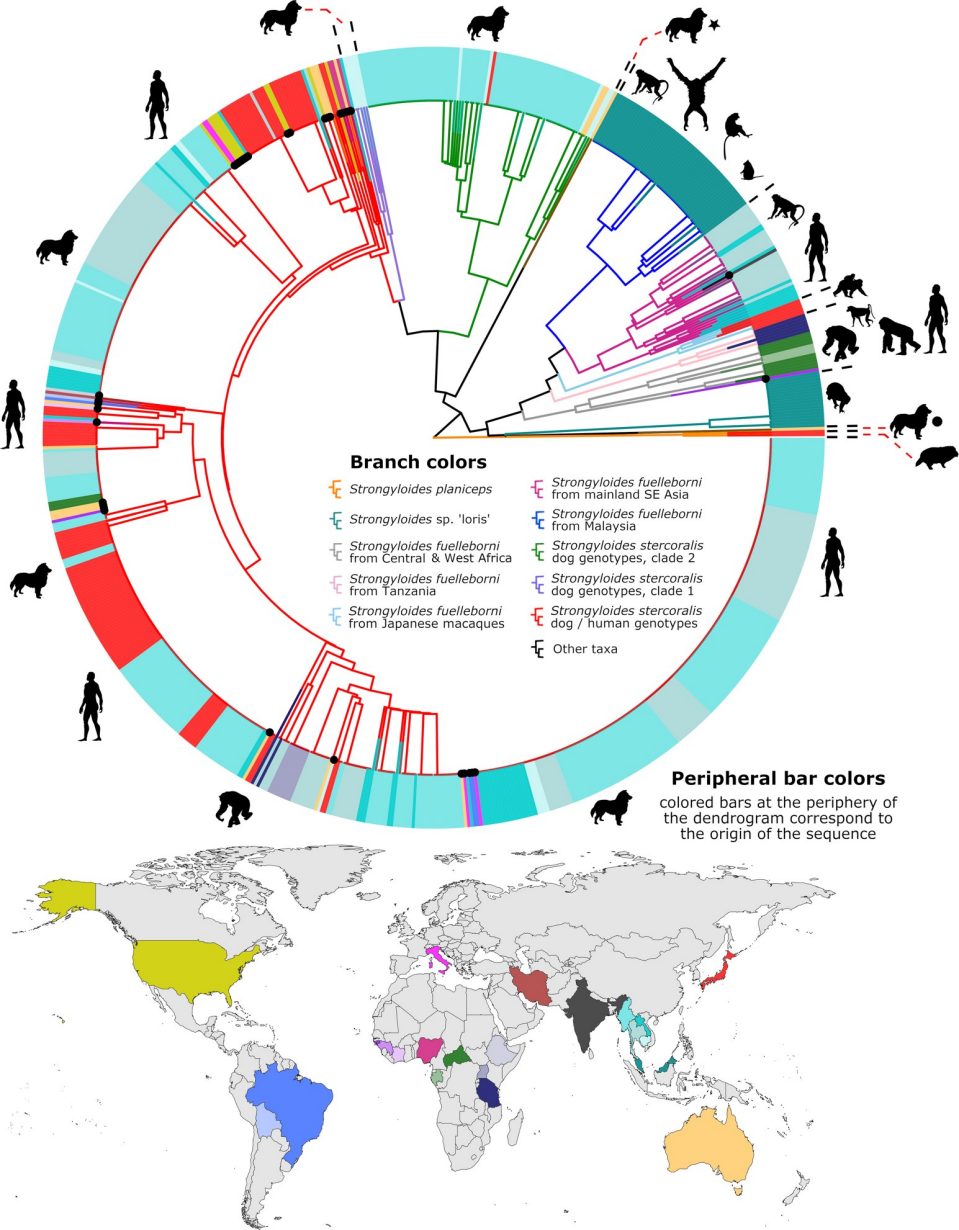
Dendrogram of clustered cox1 sequences. This dendrogram represents 787 cox1 sequences, including those generated by Barratt et al. (branches tipped in a black dot) and all published cox1 sequences from GB (at the time this figure was originally prepared) that overlap completely with our 217 base cox1 amplicon (to our knowledge). Peripheral bars are colored according to their site of origin, which corresponds to the colored countries on the map. Branches are color coded separately, according to their identity; either a species assignment, a genus, or their *S*. *stercoralis* genotype. The dog image with a black star indicates a sequence from an Australian dog generated by us previously [21], that is distinct from other *Strongyloides* spp. and clusters between the *S*. *stercoralis* and *S*. *fuelleborni* groups. The dog image with a black circle highlights a published sequence [21] that clusters close to, yet is distinct from *Strongyloides* spp. detected previously in lorises [27]. Animal images reflect the mammalian hosts that the sequences were associated with. Two sequences of *Strongyloides planiceps* (orange branches) from Japanese raccoon dogs serve as an outgroup. The identity of each sequence is provided in S1 Fig which is a searchable PDF of the same dendrogram with all GB accession numbers, the countries of origin, and host species provided. The GB accession numbers for sequences in this dendrogram that were generated as part of this study (branches tipped in a black dot) are provided in S1 File. The sequences used to construct this dendrogram are provided in S2 File. This figure incorporates all corrections detailed in Table 4.

Fig 3 is incorrect due to the error in Table 4. The authors have provided a corrected version here.

**Fig 3:**
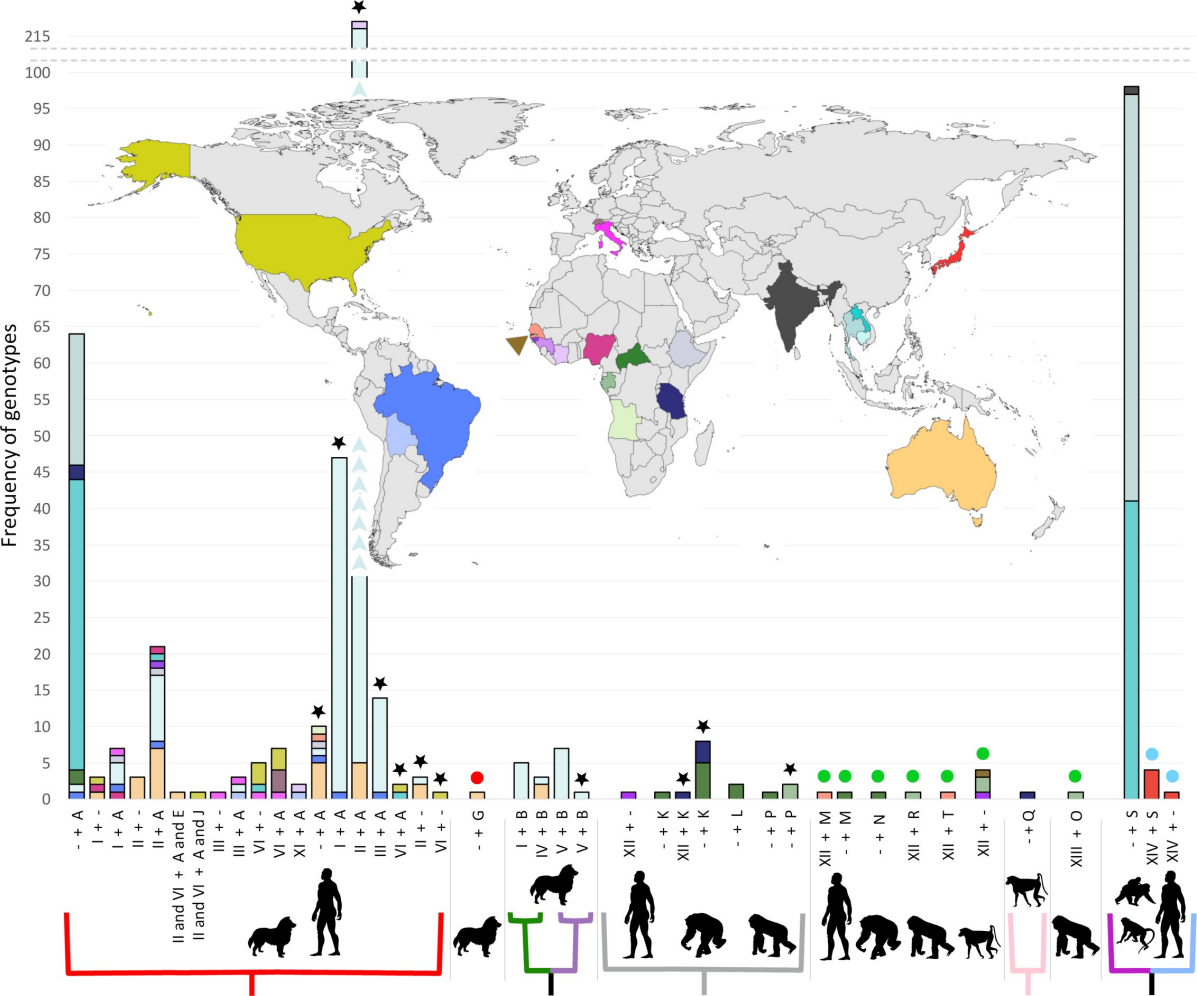
Global frequency of Strongyloides spp. 18S genotypes. Histogram bars are colored according to their origin, corresponding to the colors on the map. A dash in the horizontal axis labels (-) indicates a missing 18S haplotype (either HVR-I or HVR-IV). The colored branches below the horizontal axis correspond to the colored branches in Fig 2, where the red clade represents S. stercoralis types infecting both dogs and humans (lineage A), the green/purple clade represents S. stercoralis types infecting only dogs (lineage B), the gray clade represents S. fuelleborni types from African great apes and humans, the light pink clade represents S. fuelleborni from a baboon and the magenta/light blue clade represents types found in humans and macaques. The absence of a colored branch under a given group indicates that an associated cox1 sequence is presently unavailable for these 18S haplotypes. Black star: A cox1 sequence is not available for these specific worms yet they were assigned to their most appropriate cox1 clades based on the observation that their 18S types have only ever been found previously in worms with cox1 sequences belonging to these clades. Red circle: A novel 18S HVR-IV type without a corresponding cox1 sequence available. As this 18S type had not been described previously, its association with any particular cox1 clade is unknown and cannot be inferred. Green circles: Strongyloides fuelleborni 18S types without corresponding cox1 sequences available. As cox1 sequence data has not been provided for worms with these 18S types, their association with any particular cox1 clade is unknown. Blue circles: Strongyloides fuelleborni 18S types from Japanese macaques. Corresponding cox1 sequences are not available for these specific worms, yet they were assigned to the magenta/light blue clades because all known cox1 sequences from S. fuelleborni infecting Japanese macaques have belonged to this group. This figure incorporates the corrections detailed in Table 4.

Fig 4 is incorrect due to the error in Table 4. The authors have provided a corrected version here.

**Fig 4:**
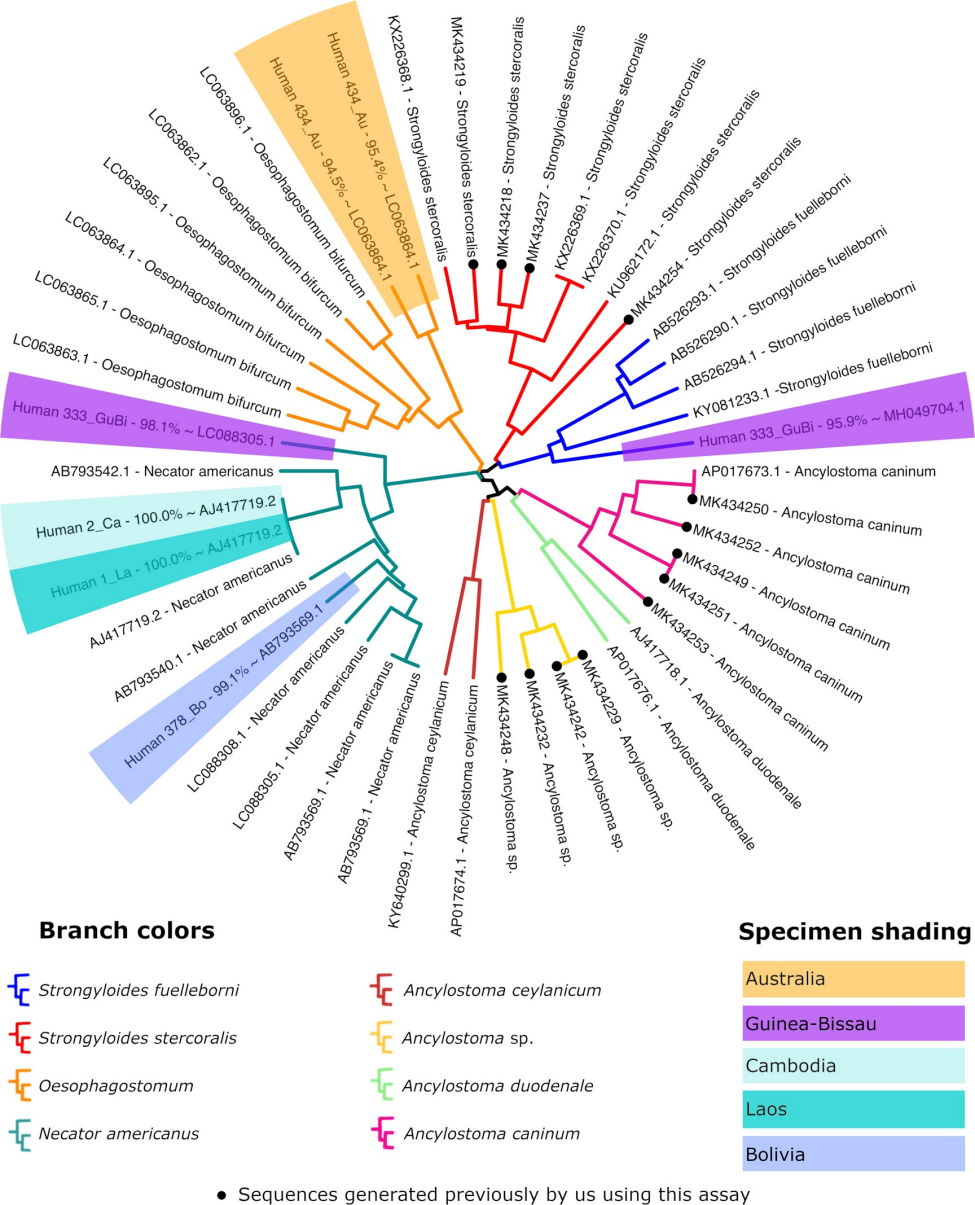
Dendrogram of cox1 sequences from *Strongyloides* spp. and hookworms. This figure demonstrates that the cox1 assay described here is broadly specific for *Strongyloides* spp. and some strongylids. Fragments of cox1 from *Ancylostoma* spp., *Oesophogostomum* and *Necator americanus* have been amplified and sequenced using this assay. Additionally, we previously detected cox1 sequences from *Metastrongylus* sp., a rotifer, and some unknown nematodes using this approach [21]. The sequences generated in this study are shaded in colors according to their country of origin and those generated by us in a previous study are marked with a black dot on the associated branch tip. The sequences used to construct this dendrogram are included in S3 File. This figure incorporates the corrections detailed in Table 4.

S1 Fig is incorrect due to the errors in the Table 4. The authors have provided a corrected version here.

S1 File is incorrect due to the errors in the Table 4. The authors have provided a corrected version here.

S2 File is incorrect due to the errors in the Table 4. The authors have provided a corrected version here.

S3 File is incorrect due to the errors in Table 4. The authors have provided a corrected version here.

## Supporting information

S1 Fig Cluster dendrogram of cox1 sequences with GB accessions numbers provided. This file serves as an aid for Fig 1 as it shows an identical dendrogram to Fig 1, yet also provides all GB accession numbers for the sequences included, along with the country of origin and host species. The file allows zooming without loss of resolution, and is also searchable for readers who wish to search the position of specific accession numbers using the ‘Find’ function in Adobe Acrobat PDF reader or another preferred PDF reader. This dendrogram incorporates the corrections detailed in Table 4.
(PDF)

S1 File Excel spreadsheet containing BioSample numbers and GenBank accession numbers. The details in this supplementary file have been updated in accordance with the corrections in Table 4. All updates are shown in red text.
(XLSX)

S2 File Fasta file of sequences used to construct Fig 2. Some of the fasta sequence headers in this file were updated according to the corrections detailed in Table 4.
(FASTA)

S3 File Fasta file of sequences used to construct Fig 4. Some of the fasta sequence headers in this file were updated according to the corrections detailed in Table 4.
(FASTA)
