# Supplementary figures and images for: Correction: A global genotyping survey of Strongyloides stercoralis and Strongyloides fuelleborni using deep amplicon sequencing
Source: PLoS Negl Trop Dis. 2021 Jun 18;15(6):e0009538. doi: 10.1371/journal.pntd.0009538 (PMC8213129; doi:10.1371/journal.pntd.0009538)

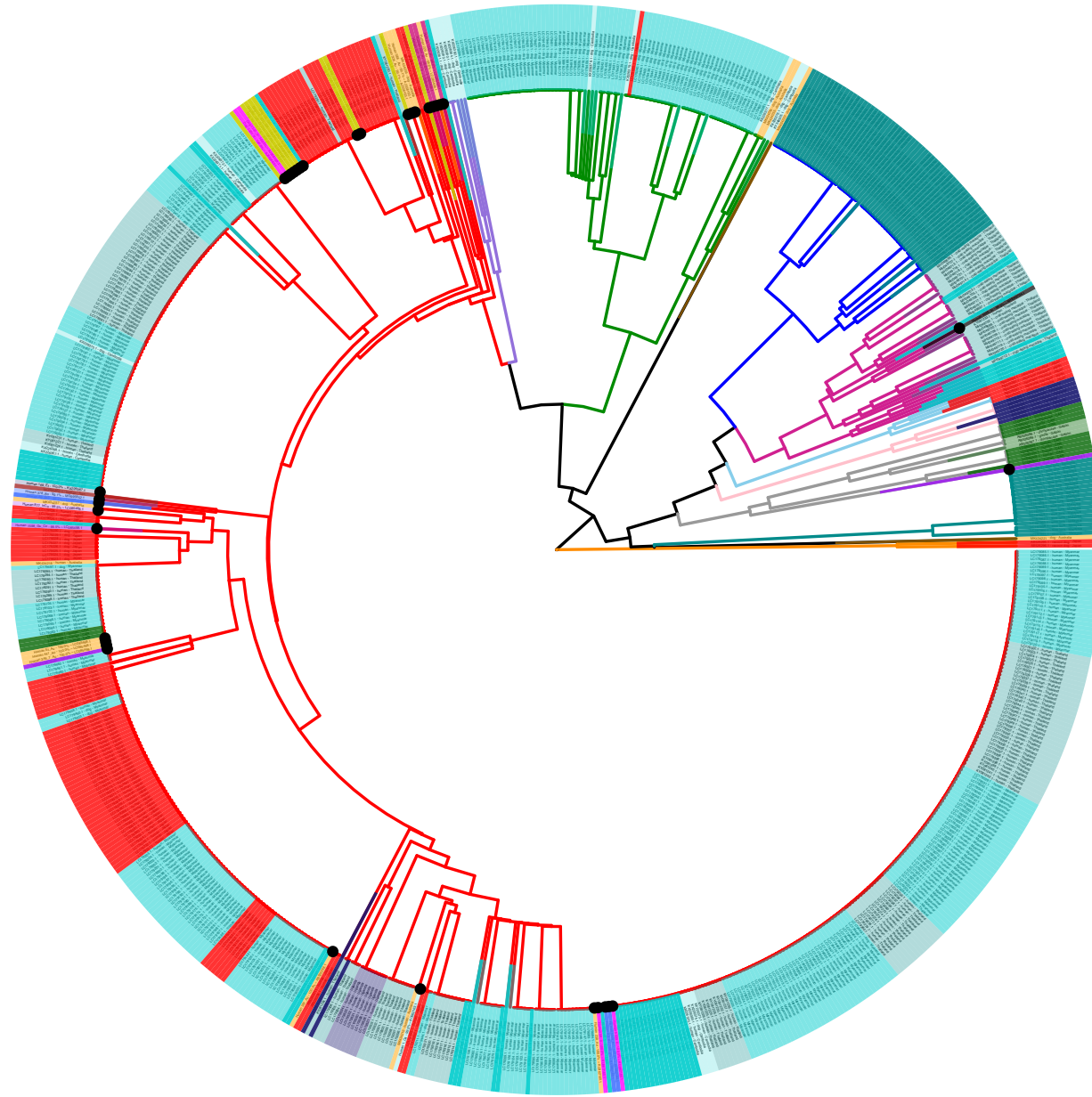

Supplement: S1 Fig — This file serves as an aid for Fig 1 as it shows an identical dendrogram to Fig 1, yet also provides all GB accession numbers for the sequences included, along with the country of origin and host species. The file allows zooming without loss of resolution, and is also searchable for readers who wish to search the position of specific accession numbers using the ‘Find’ function in Adobe Acrobat PDF reader or another preferred PDF reader. This dendrogram incorporates the corrections detailed in Table 4. (PDF) [file pntd.0009538.s001.pdf]
